# Smart chiral liquid crystal elastomers: Design, properties and application

**DOI:** 10.1002/smo.20230025

**Published:** 2024-02-29

**Authors:** Yuan Liu, Jiazhe Ma, Yanzhao Yang, Cristian Valenzuela, Xuan Zhang, Ling Wang, Wei Feng

**Affiliations:** ^1^ School of Materials Science and Engineering Tianjin University Tianjin China; ^2^ Binhai Industrial Research Institute Tianjin University Tianjin China

**Keywords:** blue‐phase liquid crystals, chiral elastomers, cholesteric liquid crystals, photonic crystals, structural color

## Abstract

Smart chiral liquid crystal elastomers are a class of soft photonic crystals with periodic nanostructures. There are two kinds of chiral liquid crystal elastomers with structural colors: cholesteric liquid crystal elastomers with a one‐dimensional helical nanostructure and blue‐phase liquid crystal elastomers with a three‐dimensional photonic crystal nanostructure. The self‐assembled nanostructure of chiral liquid crystal elastomers can be dynamically controlled under external stimulation, and the reflected color can be adjusted throughout the visible light range. Along with the development of innovative material systems and cutting‐edge manufacturing technologies, researchers have proposed diverse strategies to design and synthesize chiral liquid crystal elastomers and have thoroughly investigated their properties and potential applications. Here, we provide a systematic review of the progress in the design and fabrication of smart chiral liquid crystal elastomers, focusing on the cholesteric liquid crystal elastomers via surface‐enforced alignment, bar coating, 3D printing, anisotropic deswelling methods as well as the three‐dimensional self‐assembly of blue‐phase liquid crystal elastomers without additional alignment. Smart chiral liquid crystal elastomers are able to respond quickly to external stimuli and have a wide range of applications in areas such as adaptive optics, color‐changing camouflage, soft robotics, and information encryption. This review concludes with a perspective on the opportunities and challenges for the future development of smart chiral liquid crystal elastomers.

## Graphical abstract

In this review, we summarize the design and fabrication of cholesteric liquid crystal elastomers via diverse techniques such as surface‐enforced alignment, bar coating method, 3D printing, and anisotropic deswelling. Three‐dimensional blue‐phase liquid crystal elastomers without additional alignment are also introduced. Smart chiral liquid crystal elastomers are of paramount importance for a wide range of applications such as adaptive optics, artificial photonic skin, color‐changing camouflage, soft robotics and beyond.

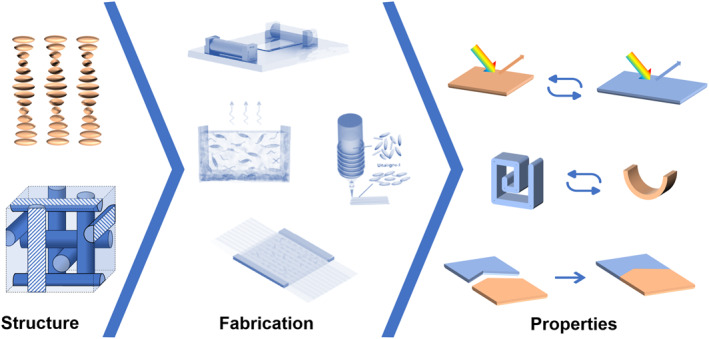

## 1 INTRODUCTION

Liquid crystals (LCs), which combine the fluidity of liquids and the ordered arrangement of crystals, have been receiving much attention since their discovery in the late 19th century.[^1^, ^2^] In recent decades, the widespread use of low molecular weight LCs in display technology has driven the rapid development of LCs in other application fields,[^3^, ^4^] including solar energy capture,^[5]^ smart photonics[^6^, ^7^, ^8^, ^9^] and biomedicine.[^10^, ^11^, ^12^] LC polymers are obtained from the polymerizing of LC monomers or by grafting LC monomers onto the main chain of flexible polymers to form a new class of functional advanced polymer materials. Based on their chemical composition and crosslinking degree, LC polymers can be divided into liquid crystal networks (LCNs) and liquid crystal elastomers (LCEs).[^13^, ^14^] The generated LCNs with side‐chain and main‐chain mesogenic units were formed by one‐step polymerization of reactive diacrylate mesogens, which were aligned in situ to form densely cross‐linked LCNs.^[15]^ Similar to low‐molar‐mass LCs, LCNs can be oriented by external factors or stimuli (such as surface‐alignment materials, shear forces, surfactants, and optical, magnetic or electric fields). These factors or stimuli help create engineered materials with complex properties and alignments after polymerization.[^16^, ^17^, ^18^, ^19^]

Most previous studies synthesized LCEs using Finkelmann's two‐step technique, which involves cross‐linking and strain‐induced alignment. This technique usually produces loosely cross‐linked films that contract in‐plane when heated.[^20^, ^21^] The stretchable properties of a LCE are similar to that of a traditional rubber: long chains of molecules that can easily slide over each other and allow the material to stretch with minimal force. The LCE can change its shape instantaneously under load because of its weak crosslinking.^[13]^ LCEs also show spontaneous orientational ordering, which allows LCEs to contract along the direction of the LC molecules due to the order‐disorder transition of mesogens.[^22^, ^23^] LCEs can undergo a phase transition in response to environmental stimuli, including temperature,[^24^, ^25^, ^26^] humidity,^[27]^ organic solvent,^[28]^ and electric field.[^29^, ^30^] Additionally, external stimuli such as light[^31^, ^32^, ^33^] and magnetic field^[34]^ can also induce the phase transition. By controlling molecular orientations, LCEs can accomplish contactless movement in response to different environmental inputs. LCEs have high chain anisotropy and greater reversible deformation when exposed to external stimuli.^[35]^ These properties make them widely used in the fields of soft robots,[^36^, ^37^, ^38^] artificial muscles,[^39^, ^40^] optical devices,[^41^, ^42^, ^43^] and self‐regulating devices.[^44^, ^45^]

Chiral liquid crystal elastomers are a type of LCE with a specific helical nanostructure. The structural colors of chiral liquid crystal elastomers are caused by the helix nanostructure rotation of the LCE directors, which makes them attractive as photonic films.^[46]^ Chiral liquid crystal elastomers such as cholesteric liquid crystal elastomers (CLCEs) and blue‐phase liquid crystal elastomers (BPLCEs),^[47]^ can be self‐assembled by introducing chiral molecules into LCEs.[^48^, ^49^] CLCEs have a one‐dimensional chiral helical nanostructure and an elastomeric network, which reflects circularly polarized light with the same handedness as the helical nanostructure.^[50]^ The coupling between the cholesteric helix and the elastomeric network causes the reflection color and polarization to change with mechanical deformation.[^51^, ^52^] The helical nanostructure and the dimensions of CLCEs can respond to mechanical stress leading to a change in the reflected color.[^53^, ^54^] Therefore, CLCEs have a variety of potential applications in optical sensors and devices.^[55]^ Chiral liquid crystal elastomers with blue phase are materials with cubic lattice nanostructures that form 3D chiral photonic crystals. These photonic crystals can control the flow of photons or light in all directions.[^56^, ^57^] 3D blue‐phase liquid crystal (BPLC) molecules form double‐twisted cylinders by arranging themselves in a double‐twisted state, such that cylinders stack on top of each other to create two kinds of periodic 3D lattices. The lattices have either body‐centered cubic symmetry or simple cubic symmetry, and they show a narrow photonic band gap.[^58^, ^59^, ^60^] The creation of intelligent optical devices that respond to stimuli, such as powerful 3D tunable lasers, would be propelled by the continuous advancement in the manipulation of BPLCEs.[^61^, ^62^] These BPLCEs can selectively reflect circularly polarized light due to their chiral photonic nanostructures, and also respond sensitively to stimulus changes such as force, heat, electricity, light, humidity, etc., while exhibiting dynamic modulation of structural colors, which has wide application prospects in the fields of color changing camouflage, information encryption, tunable laser, etc.[^63^, ^64^, ^65^, ^66^, ^67^, ^68^, ^69^, ^70^] It should be pointed out that several reviews have summarized the recent advancement of chiral liquid crystals, LCEs and their technological applications.[^49^, ^71^] Recently, diverse alignment methods including surface‐enforced alignment, bar coating, 3D printing and anisotropic deswelling have been developed to develop chiral liquid crystal elastomers with stimulus‐responsive structural colors, broadband spectral shifts, good mechanical properties, and large‐scale processability, and identified their potential applications in biomimetic camouflage, information encryption, and programmable shape. However, there remains a notable gap in the literature for a comprehensive review encompassing the design, properties, and applications of chiral liquid crystal elastomers. To provide useful insights to the research community, it is necessary to summarize the recent progress related to chiral liquid crystal elastomers.

**FIGURE 1:**
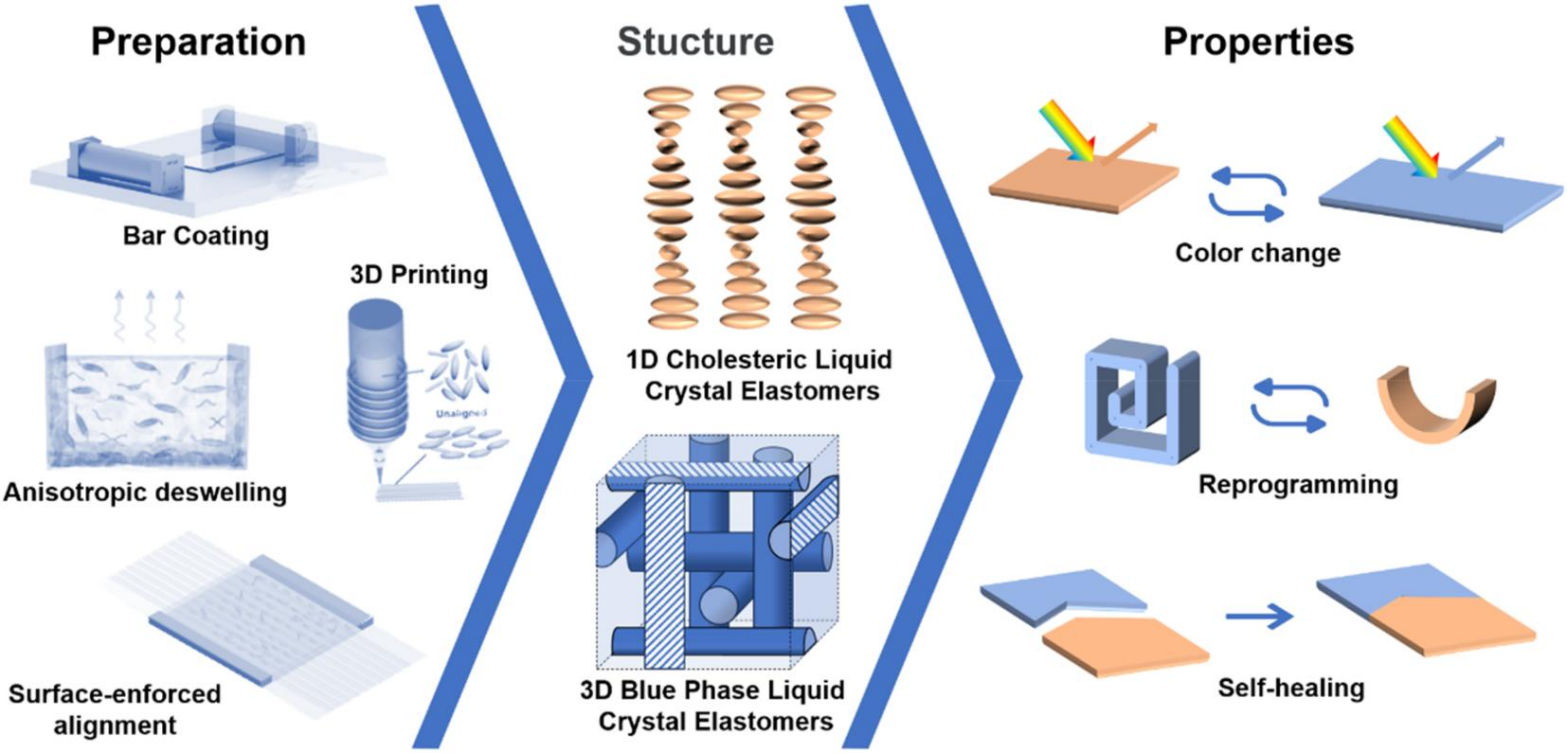
Preparation structure and properties of chiral liquid crystal elastomers with color change, self‐healing, and re‐programmability.

Herein, we provide a comprehensive review of the research state‐of‐the‐art advances of applying chiral liquid crystal elastomers in the fields of alignment methods. We systematically discuss the recent design and self‐assembly strategies such as the surface effect‐induced alignment method, bar‐coating method, 3D‐printing method, and anisotropic deswelling method for the self‐assembly of CLCEs and BPLCEs, and how these methods have been applied to create functional materials as well as their applications in biomimetic camouflage, information encryption, and shape‐programmable (Figure 1). Finally, the challenges and future development direction of chiral liquid crystal elastomers are further discussed. A summary of the scattered literature in this emerging research field is expected to attract more attention of researchers from multidisciplinary fields and prompt the development of chiral liquid crystal elastomers towards diverse applications such as soft robotics, information encryption, mechanical metamaterials, and other bioinspired artificial intelligent systems.

## 2 CHOLESTERIC LIQUID CRYSTAL ELASTOMERS VIA SURFACE‐ENFORCED ALIGNMENT

The surface‐enforced alignment method usually consists of preparing a LC cell whose glass surface has been subjected to treatments to impose a certain mesogen orientation direction (e.g. surface rubbing or photolithography).[^72^, ^73^, ^74^, ^75^] LC monomers are firstly both reacted with chain extenders and a catalyst in a given ratio to produce oligomers and mixed directly with photoinitiators to then inject into the cell by capillary force. When the mesogens interact with the alignment coating and anchor near these microscopic and patterned surfaces, chiral agents induce spatial variation of the molecules and cause self‐assembly of a helical structure. The topographically aligned mixture is then fixed by UV light or thermally‐initiated radical polymerization, resulting in the formation of a cross‐linked main‐chain CLCE.

The surface‐enforced alignment method was employed by White et al. to produce well‐oriented (single domain) CLCEs. The LC mixtures were polymerized within alignment cells with planar alignment, resulting in comparatively homogenous pitch distributions that enhance the optical quality of the polymeric films. By doing this, they can electively control the reflection wavelength in the visible range based on temperature. It also creates a strong thermomechanical coupling that responds to a wide range of stimuli. The findings demonstrate the distinctive potential of implementing LCEs to control light reflection in diverse fields including textiles, optics, and architecture.^[76]^

The fabrication of programmable CLCEs is challenging. Bowman et al. designed a flexible, programmable, and mechanochromic CLCE by gently shearing a viscous CLC oligomer between glass slides to create a reflective surface in minutes. When the CLCE is strained, its color shifts blue through the visible spectrum, and when the material is heated to its isotropic phase, it recovers its original color and shape. The activation of the dynamic covalent bonds in the chiral liquid crystal elastomers via light exposure transforms reversible strain and color changes into permanent states at any chosen strain, while subsequent heating can temporarily revert the shape and color to nearly their initial state. Thanks to the dynamic covalent chemistry character, the material was patterned to mimic chameleon skin and displayed strain mapping capabilities.^[77]^

Although surface‐enforced alignment is a method that improves without requiring CLC systems or delicate equipment setups, it also has some drawbacks, such as creating static charges, dust, and scratches on the surface of alignment. To solve this problem, Yang and coworkers prepared and programmed nematic LCEs with 1D microchannel patterns by combining photolithography and soft lithography techniques. By patterning more complex LC director profiles, researchers were able to precisely control the LC alignment and generate a wider range of folded LCE forms. This strategy thus enables the miniaturization of LCE actuators and boost advances in applications such as microrobots, sensors, and artificial muscles with large work capacities.^[78]^

The multilayer concept enables the design of CLCEs with dynamic mechanical response and desired material properties depending on the orientation patterns of mesogens, as proposed by Tsutsumi and colleagues.^[51]^ By simply changing the external layers, polydimethylsiloxane (PDMS) or polymethylpenten (PMP), the researchers discovered the potential to control the 3D orientation direction of molecules during deformation. Such as CLCEs/PDMS (rapid) and CLCEs/PMP (ultraslow) show promise in the development of mechano‐optical sensors. CLCEs/PMP can fix color after removing force, which is suitable for strain‐memory applications. CLCEs/PDMS can be sensed in real‐time, which is useful for soft robotics and motion sensing. They proposed a fundamental concept for developing methods to easily tune and control the mechanical properties of LCEs without modifying the molecular structure.

**FIGURE 2:**
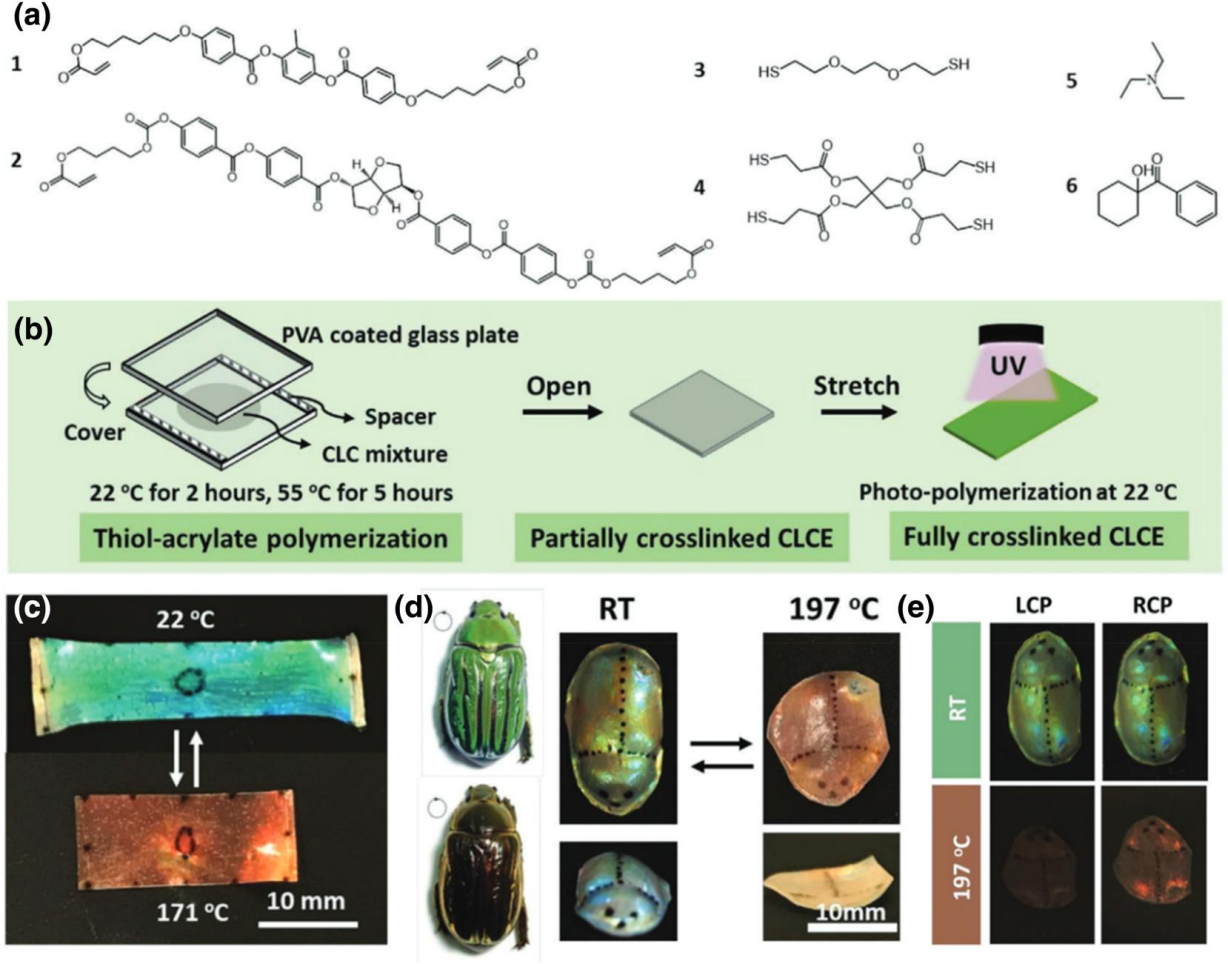
(a) Chemical elements of the materials used in the creation of CLCE mixtures. (b) The two‐step process of crosslinking to create CLCE films that respond to temperature (c) Images of the CLCE film taken at various temperatures. (d) Photographs of the beetle C. gloriosa viewed with left‐hand circular polarizers (LCP) and right‐hand circular polarizers (RCP). (e) Images of CLCEs that have been programmed and viewed at several temperatures using LCP and RCP.^[79]^ Copyright 2021, Wiley‐VCH.

A surface‐enforced alignment method was proposed by Schenning et al. to fabricate 3D‐shaped CLCE structural color actuators that can switch their hyper‐reflectivity.^[79]^ CLCE was fabricated via a two‐step crosslinking method that included a base‐catalyzed thiol‐acrylate reaction and a light‐induced free radical polymerization reaction. As shown in Figure 2a, the CLCE mixture consists of 6 components, which were blended and melted together, and then allowed to cool down to room temperature (≈22°C). A drop of the mixture was applied onto a polyvinyl alcohol (PVA)‐coated glass substrate with pieces of adhesive tape attached to the edges as spacers and then covered with a second PVA‐coated glass slide to form a cell. After shearing the glass substrates in a single direction to align the mixture, the reaction was conducted at 22°C for 2 h and then at 55°C for 5 h to speed up the cross‐linking process (Figure 2b). To obtain a freestanding film, the sample cell was soaked in deionized water at 50°C for several hours to dissolve PVA and peel the film off the glass substrates. The film was then mechanically deformed and photo‐polymerized to program the desired shape, color, and reflectivity. As illustrated in Figure 2c, the programmed CLCE film can reversibly alter its color and shape between the programmed and original shapes by temperature driving. Using the moulding method, the authors have prepared a 3D beetle model with circularly polarized structural colors, as shown in Figure 2d,e. The CLCE bionic beetle is able to change its shape, color, and circular polarization reflections simultaneously when the temperature is changed. This is because deforming the helical nanostructure results in a deformed helical pitch, which reflects circularly polarized light from both the left and the right sides. After heating, the film recovers its helical nanostructure as well as its polarization selectivity, which also changes reversibly from reflecting equal amounts of left and right polarized light to reflecting only right polarized light. This paper provides a straightforward technique that can be used to develop arbitrary programmable and reversible 3D shapes and photonic reflections, benefiting numerous industries, including sensors, actuators, and switchable super‐reflective materials.

Brighter color, environmental friendliness, and longer lifetime are among the benefits of structural color films. However, the sample's geometric constraints and physical size limit the tunable range of the structural color, making broadband pixelated color switching challenging to produce. A considerable degree of freedom may be achieved in the arrangement of the pixels and air channels, and the special mechanochromic properties of CLCE will encourage the development of complex, highly responsive, and bio‐inspired photonic devices. Hence, Yang et al. used a two‐step process to develop a gas‐driven CLCE film by first stabilizing an LC prepolymer with a chiral nematic solvent and then photopolymerizing it followed by removing the chiral nematic solvent.^[80]^ Despite the relatively basic procedures, the exceptional mechanochromic performance of CLCEs and the extensive degree of flexibility in the design of the pixels and air channels. These features could enable the development of biomimetic, highly responsive, and complex photonic devices.

**FIGURE 3:**
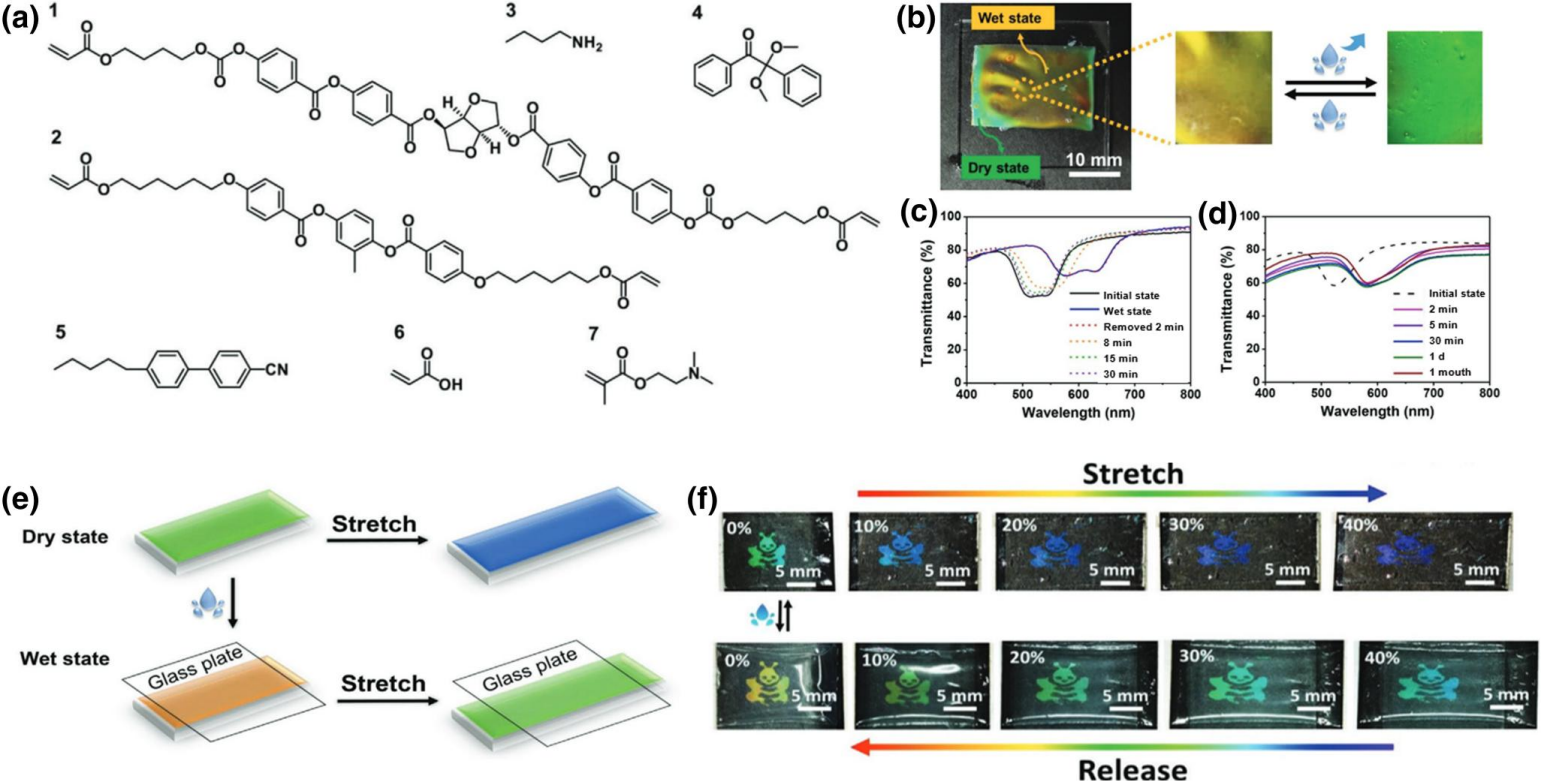
(a) Chemical conformations of the mixture used to prepare of the CLCE‐poly(ampholyte) SIPN‐PDMS bilayer film. (b) Images of the bilayer film before and after it was wet. (c) Time required for water to evaporate from the bilayer film at room temperature. (d) The bilayer film is subjected to varying durations of immersion in deionized water to determine its stability. (e) Diagrammatic representation of the bilayer film's structural alterations during stretching in wet as well as dry states. (f) Bilayer film patterned with a bee in both dry (top) wet (bottom) states, actuated using reversible mechanochromic processes.^[81]^ Copyright 2021, Wiley‐VCH.

Zhou et al. proposed a new strategy to fabricate a dual‐responsive elastic cholesteric polymer material with tunable chiral properties. Using the surface‐enforced alignment technique, they created a semi‐interpenetrating network (SIPN) consisting of a CLCE and a hydrophilic poly(ampholyte) network.^[81]^ Figure 3a shows the chemical structures of each component that makes up the color‐changing dual‐responsive material. Since the introduced hydrophilic polymer is capable of swelling in water, this volume change can cause a color change in the CLCE‐poly(ampholyte) SIPN‐PDMS bilayer film by increasing the pitch, causing a red shift from green to orange (Figure 3b). The original visible state was recovered after the water evaporated. The bilayer film's reflection spectra in both wet and dry states were obtained to assess the hydrochromic behavior and the time needed for the water to evaporate at room temperature (Figure 3c). Compared to previous studies, the wavelength of the reflection band was shown to increase by 11%, and have a very stable reflection band even after 1 month of exposure to water (Figure 3d). Furthermore, the water‐ and strain‐induced color changes are independent of each other, since the amount of red shift caused by the presence of water is maintained when deformation is applied compared to the stretched and dried film (Figure 3e). To prevent the water from evaporating from the networks, a small glass plate was placed on top of the film until the bilayer film reached swelling equilibrium. This finding also demonstrated that when the material was subjected to both water and a mechanical strain of around 30%, patterned responsive photonic films had the same reflection color as the dried patterned bilayer film (Figure 3f). The material achieves increased functionality by maintaining the elasticity and responsiveness of the main‐chain polymer while also acquiring responsiveness from the second polymer. The material's flexible and non‐brittle properties facilitate its use as a versatile wearable device, creating new opportunities for developing wearable optical devices and sensing applications. In this section, we summarized CLCEs prepared using the surface‐enforced alignment method and demonstrate their applications in cryptography, adaptive optics, soft robotics, and mechano‐optical sensors.

## 3 CHOLESTERIC LIQUID CRYSTAL ELASTOMERS VIA BAR COATING

During the bar‐coating process, the LC molecules are aligned along a common direction due to the shear and drag forces between the viscous LC mixture and substrate, forming a planar CLC alignment layer, and observing a bright circular polarization with reflective colors within the visible light range, without the need for additional upper alignment layer or surfactants. The substrate is first heated to a specific temperature, and then a specific amount of LC precursor mixture is added, which is spread evenly over the substrate by sliding the bar‐coater at high speed and followed by photopolymerization to create a cross‐linked CLCE film. In this method, it is possible to control the film thickness by adjusting the gap height, so it can be applied to the preparation of photonic reflective coatings while reducing the number of subsequent coating layers. Schenning et al. presented this method to prepare reflective photonic bilayer coatings that effectively adhere to plastic substrates by using a type II photoinitiator to trigger covalent bonding at the interface.^[82]^ In a later study, the authors aligned a CLC mixture by bar‐coating before initiating a two‐step thiol‐acrylate polymerization using a method to prepare elastomeric coatings with CLC phase, which can reflect specific wavelengths of light.^[83]^ By monitoring the structural color changes of the CLC coating film, they could predict and program the molecular weight between crosslinks and the crosslinking density of the CLCE network. They also demonstrate how to create multicolored patterns, broadband coatings, and dual‐responsive coatings that can change color with solvents and temperature. These coatings are important for the large‐scale production of broadband reflective photonic crystals.

A cross‐linkable main‐chain CLC oligomer was used by Zhang and colleagues to develop a simple and versatile technique for making reflective patterned coatings that respond to temperature changes by photo‐crosslinking specific areas at different temperatures using the bar‐coating method.^[84]^ In further work, the researchers fabricated a free‐standing patterned mechanochromic photonic bilayer film by using a photomask to locally crosslink the CLCE layer and by laminating a soft PDMS layer on top of it. Photonic bilayers have great potential for various applications, such as sensors that visualize strain and bending deformation, data encryption that records diverse information, or anti‐counterfeit measures.^[85]^

**FIGURE 4:**
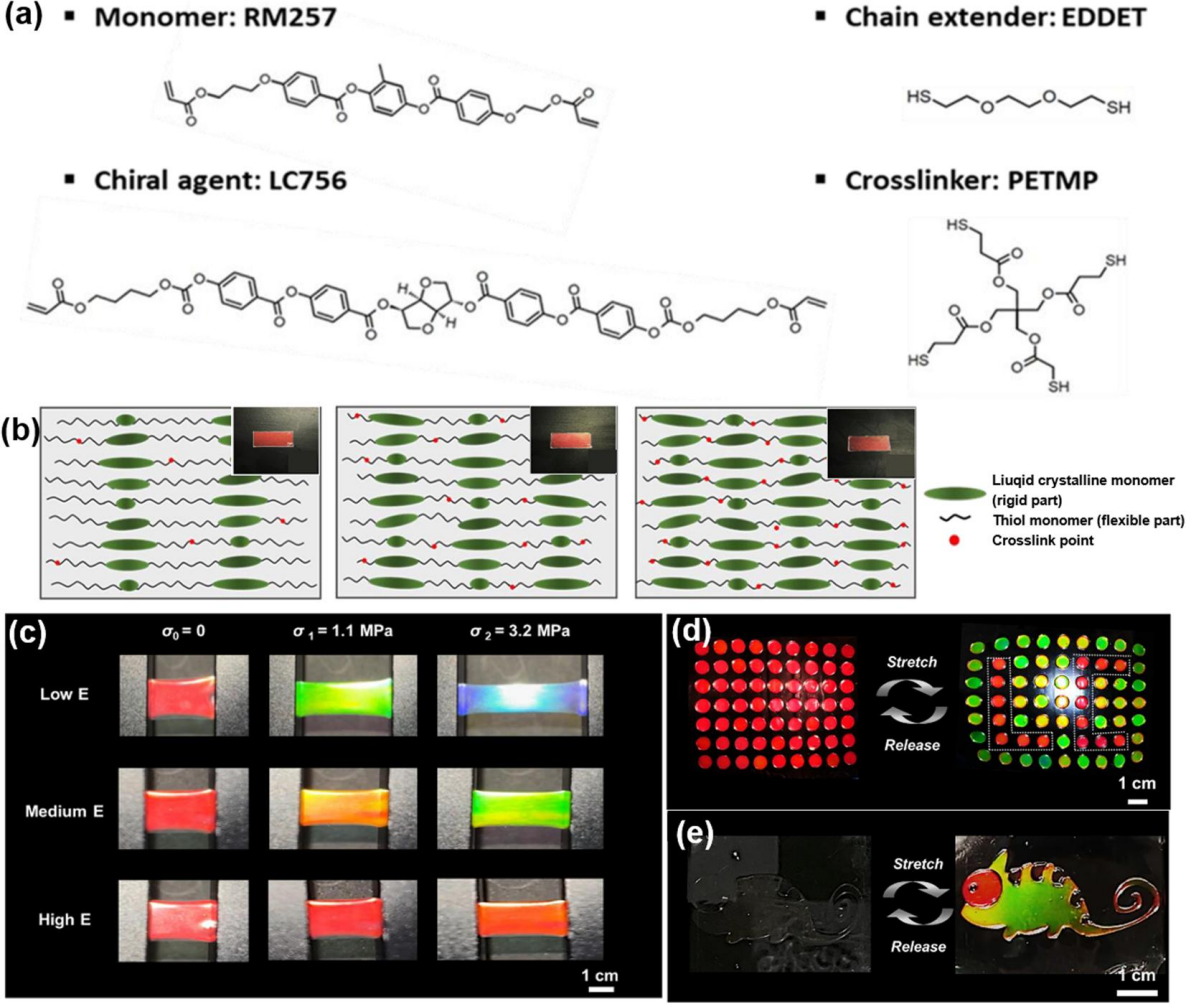
(a) Chemical composition of the starting materials used to create stretchable CLCE precursors. (b) Initially reflection color and crosslinking density schematic for CLCEs with low, medium, and high elastic moduli. (c) Images of CLCEs illustrating the separation of reflection color under constant tensile strain of *σ*_0_ = 0, *σ*_1_ = 1.1 MPa, and *σ*_2_ = 3.2 MPa. (d) Stretchable color switching with “LC” camouflaged information. (e) Multi‐color changing, invisible chameleon photonic e‐skin control.^[86]^ Copyright 2023, Wiley‐VCH.

Choi et al. employed bar‐coating to deposit a chiral nematic LC oligomer solution onto a flexible substrate and then crosslinked when the self‐assembled photonic structure reflected the desired color.^[86]^ Multicolor separation using mechanical or electrical stretching using engineered modulus‐adjusted CLCEs was then investigated. Figure 4a,b illustrate how the elastic modulus of the CLCEs can be controlled by changing the excess acrylate concentration of the precursor mixture. As expected, under the same tensile stresses of 1.1 and 3.2 MPa, distinct wavelength shifts were observed for each CLCE due to their different elastic moduli (Figure 4c). Therefore, simultaneous stretching of CLCEs with different modulus values results in individually different strains and wavelength responses, which allows the separation of initially single‐red CLCEs into multiple colors. To investigate the potential of CLCEs for disguising and camouflaging applications, the authors further developed a series of stretchable letters and chameleon‐like photonic patterns to achieve invisibility control based on multicolor separation switching (Figure 4d,e), which enhances the functionality of numerous possible photonic applications.

**FIGURE 5:**
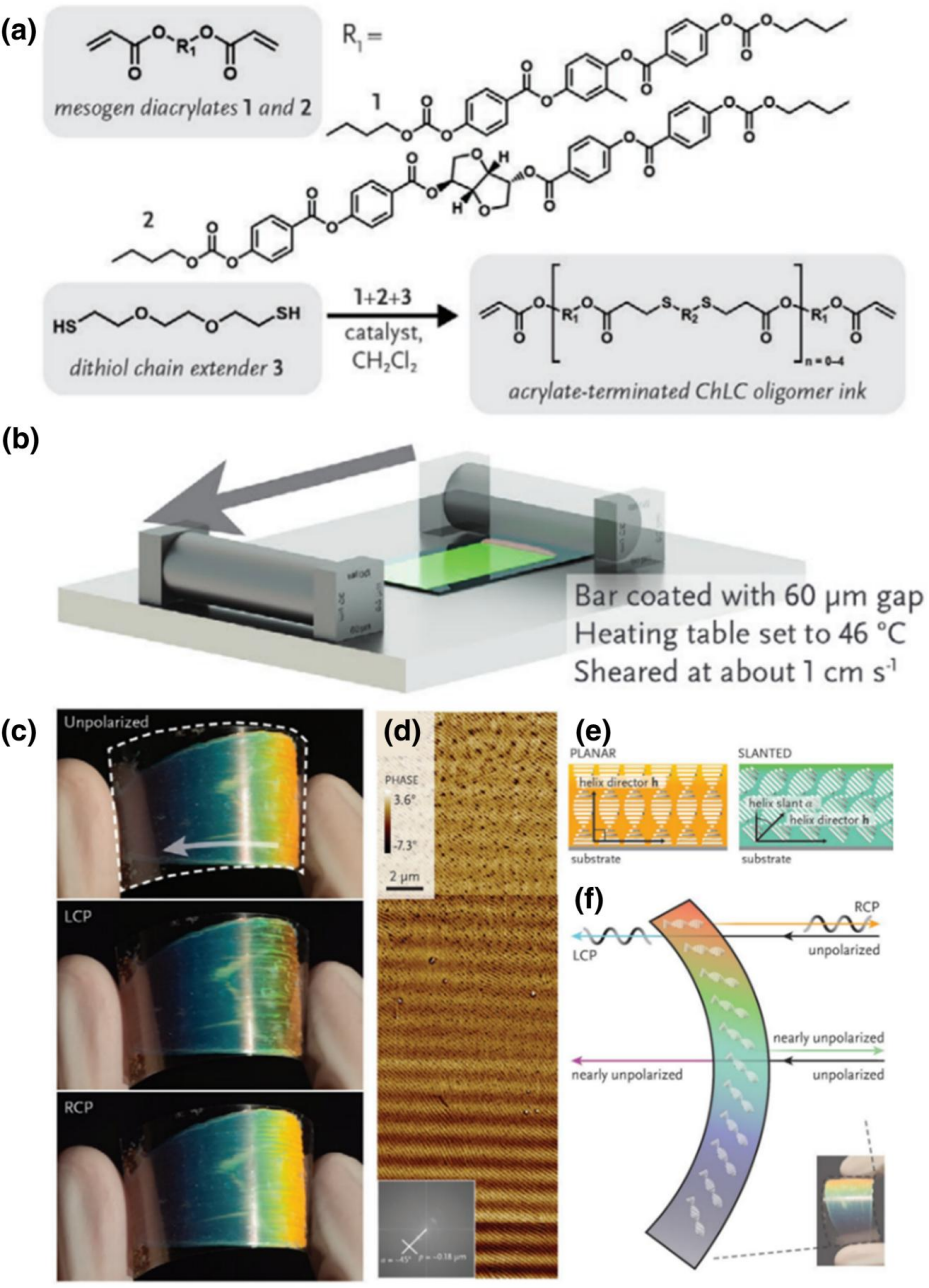
(a) Chiral nematic LC oligomer ink's main constituents shown schematically. (b) Program illustration of the bar‐coating process. (c) Flexible plastic substrate polymerized and bar‐coated with crosslinked photonic film. (d) Atomic force microscopy images showing the nanoengineered slanted photonic nanostructures. (e) Schematic illustrating the molecular alignment in a suggested slanted photonic nanostructure (right) and a traditional chiral nematic reflector (left). (f) Schematic illustrating the object's optical response.^[87]^ Copyright 2021, Wiley‐VCH.

Chiroptical properties can be observed in nature and allow selective interactions with specific wavelength bands of specific polarization states. However, forcing the planar chiral nematic into a slanted configuration to fabricate chiroptical diffraction elements is no easy task, especially when one wants to distribute these interactions over the surface of a single object. Recently, the researchers synthesized and characterized a CLC ink that can self‐assemble into a slanted photonic structure with tunable optical properties. The major molecules for the thiol‐acrylate Michael addition oligomerization of the chiral nematic LC oligomer ink are schematically represented in Figure 5a. The CLC oligomer ink was then melted onto a pre‐treated glass substrate and bar‐coated at 46°C (Figure 5b), immediately giving rise to a coating film whose peak reflected wavelength *λ*_max_ is not viewed from the surface normal, as expected for a chiral nematic coating. The distorted helicoidal structure was thus suggested to arise from the viscosity of the ink in contrast to the planar cholesteric nanostructures formed from bar‐coated diluted CLC inks. As seen in Figure 5c, photoinduced crosslinking creates a soft rubber CLCE coating that maintains the unusual photonic structure, while Figure 5d shows the AFM image of the cross‐section of the material, which reveals a slant angle of about 45° and a cholesteric half‐pitch of 0.18 μm. In Figure 5e, the molecular alignment scheme for a traditional chiral nematic reflector (left) and a tilted photonic reflector (right) are depicted, which explain the difference in their optical response. Depending on the angle of observation and illumination, the material reflects different colors and polarizations, as depicted in Figure 5f. The authors further provided an experimental basis for translating bar‐coating helicoidal alignment to a higher degree of spatial control using 3D printing, thus demonstrating the potential applications of this material for biomimetic optics, anticounterfeit markers, decorative coatings, and optical signaling features.^[87]^ The authors further provided an experimental basis for translating bar coating to a higher degree of spatial control using CLCE 3D printing. In addition to simple processing steps, bar coating also exploits the self‐assembling capacity of CLCEs, allowing for scalable fabrication of large‐area and roll‐to‐roll polymer devices and rapid prototyping, which has been widely used in specialized anticounterfeit markers, multi‐color concealed camouflage switching and other fields.

## 4 CHOLESTERIC LIQUID CRYSTAL ELASTOMERS VIA 3D PRINTING

3D printing is a promising high‐throughput and scalable technology for the future of advanced manufacturing, and it has been applied in areas such as tissue engineering, electronic devices, and high‐performance super materials.[^27^, ^88^, ^89^] Two common 3D printing techniques to fabricate LCEs are Direct Ink Writing (DIW)[^90^, ^91^, ^92^, ^93^] and Digital Light Processing (DLP).[^33^, ^94^, ^95^, ^96^] DIW printing creates a 3D structure by extruding non‐crosslinked viscous LC oligomer inks from a printing nozzle along the x‐ and y‐directions following a computer‐designed pattern. During extrusion, shear forces cause the LC molecules to align along the printing path, forming monodomain orientations, and the macroscopic printed object is subsequently UV photo‐crosslinked to fix the alignment.^[97]^ The extrusion‐based DIW printing is, however, limited by the printing time, which varies depending on the part size and resolution, and consequently restricts the geometric complexity of the 3D object. DLP‐based 3D printing is a process where photocurable LC mesogens are successively and efficiently printed in a layer‐by‐layer fashion, followed by localized photopolymerization to lock in large‐scale 3D soft materials with high‐resolution and complex structures. Due to its built‐in shear separation mechanism (also known as the minimum force mechanism), the LC mesogens are aligned to the backbone of the flexible polymer chains by the shear force applied to the LC resin as it slides over each thin layer (20 μm). A low‐modulus main‐chain LCE is thus formed when the polymer chains are cross‐linked and polymerized within the relaxation time scale by projected UV patterns which fix the in‐plane orientational order. Li et al. reported a DLP process for layer‐by‐layer automatic shearing of LC oligomers to produce LCE soft robots with high energy density and high orientational order.^[33]^ This printing technique was exploited by Yakacki et al. for the rapid prototyping of ultra‐lightweight 3D energy‐absorbing structures with a high level of resolution and exhibiting greater strain‐energy dissipation compared to those printed from a commercially available photocurable elastomer resin.^[95]^ Besides printing energy‐dissipative lattice structures from soft materials, the development of printed LCE actuators is another key application area. Cai et al. used heating to vary the stiffness of different regions within DLP‐printed LCEs. They controlled the printing temperature, material stiffness, and shrinkage capacity/actuation of these materials.^[98]^ Xie et al. described a DLP process for the ultrafast template‐free manufacture of LCE artificial muscles that can perform complex movements with designable patterns.^[96]^ They used natural light attenuation in the through‐plane direction to induce mesogen alignment for reversible bending action.

Kotikian and coworkers used DIW to produce a soft actuator fiber composed of a liquid metal core surrounded by an LCE shell.^[99]^ This core‐shell 3D printed design not only solves the problem of mechanical mismatch between the heating element and the LCE but also enables closed‐loop control and the sensing of external stimuli according to the change in resistance during the response process. Qi et al proposed a unique approach for on‐demand 4D printing of freestanding LCEs with an actuation strain of up to 40% using laser‐assisted DIW.^[94]^ This technology was further hybridized with the DLP method to produce active 3D structures in a single additive phase with alternative structural or movable supports. The combination of functionally freestanding LCEs printed by DIW and the supporting structures printed by DLP offers new design and production opportunities for applications such as intelligent structures, active metamaterials, soft robotics, and intelligent wearable technology.

**FIGURE 6:**
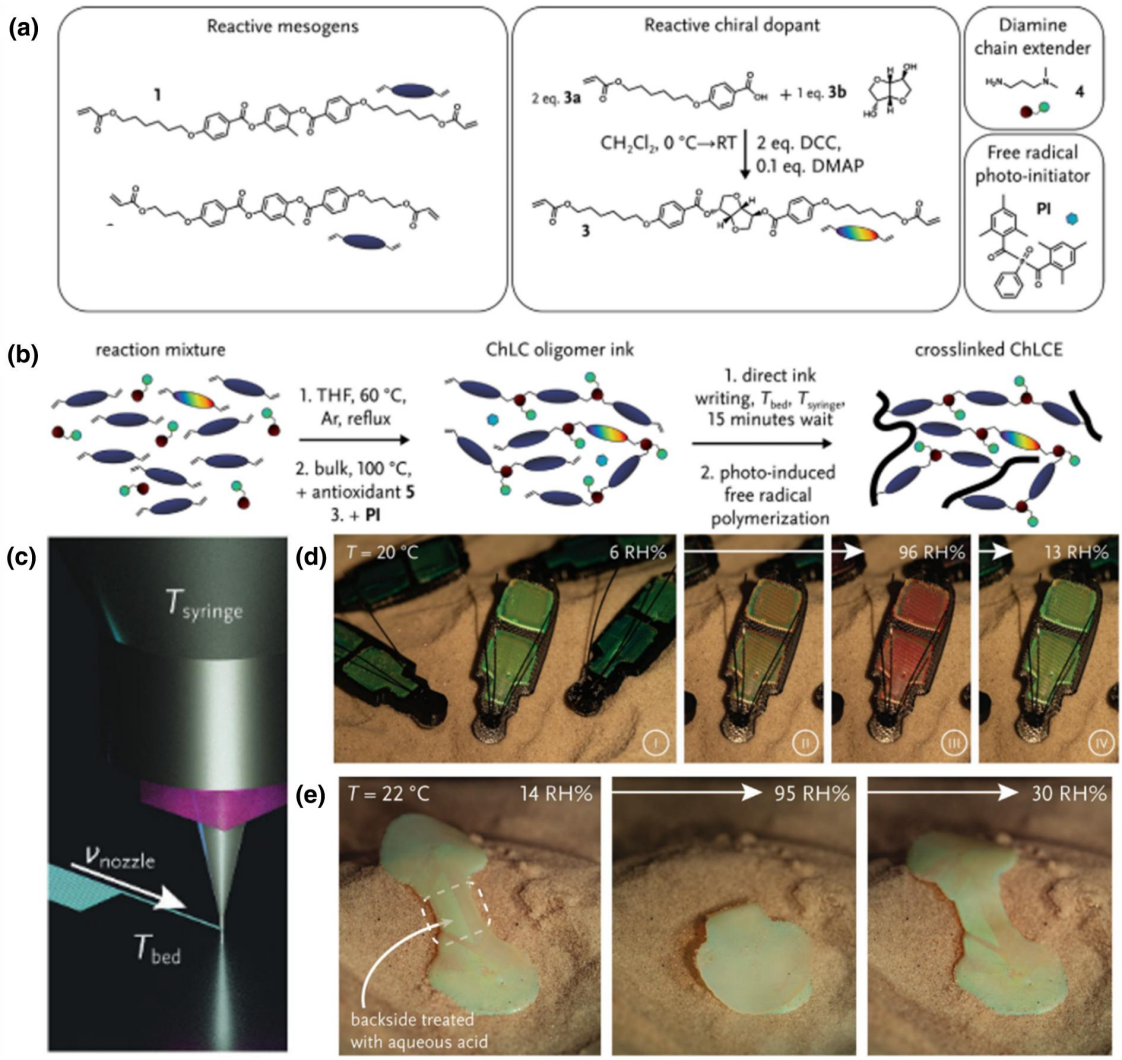
(a) The chemical components of the elements used to create the cholesteric liquid crystal (CLC) oligomer ink. (b) Schematic drawing of the molecular composition of the CLC precursor mixture before the chain extension reaction, following oligomerization, and applying acrylate crosslinking. (c) Key processing parameters for DIW optimization. (d) A group of images displaying a colony of water‐responsive 3D‐printed beetles at varying relative humidity levels. (e) Demonstration of the actuation of a bioinspired scallop at 22°C with increasing, after which the relative humidity decreased (14–95–30 RH%).^[100]^ Copyright 2022, Wiley‐VCH.

Given that chiral nematic LC oligomer inks possess similar chain extension and rheology features to those of LC oligomer inks, the shear and elongational forces of 3D printing technologies have been successfully applied to generate bright, selectively reflecting prints. In this regard, the DIW printing technique has been widely exploited to print 3D‐ and 4D‐printed structurally colored objects. Debije et al. developed stimuli‐responsive printed materials by versatile forming water‐responsive CLC oligomer inks with amine groups as pendant chain extenders (Figure 6a).^[100]^ After processing parameters for DIW, CLC inks were deposited, photo‐crosslinked, and lastly protonated to create hygroscopic ammonium groups in the polymer backbone, thereby fabricating humidity‐responsive photonic soft actuators with multicolored appearance. CLC oligomer inks with water reactivity can be used to create photonic, humidity, and reactivity actuators with multicolored appearance. A structurally colored actuator was designed by 3D printing a humidity‐sensitive CLC oligomer ink. Figure 6b shows the molecular makeup of the CLCE max before the chain extension process and after acrylate crosslinking and oligomerization. Figure 6c shows a diagram of DIW ink direct writing. The material absorbs water and swells as the relative humidity increases, causing a red‐shift of the reflected color from green to red (Figure 6d). A bioinspired structurally colored scallop‐shaped actuator was also printed, and after localized protonation treatment, a humidity‐responsive hinge emerged between the shells, creating asymmetric swelling properties that reversibly “open” and “close” the shells, which had two shells with complementary colors. Acid was precisely applied to the hinge region between the shells on the exterior, creating asymmetric swelling properties in water. This enabled the shells to “open” and “close” reversibly when exposed to dry and moist air, respectively (Figure 6e). In this case, the structurally colored actuators showed no color change when actuated. This versatile CLC ink can therefore be used to design 3D objects that can change their shape, color, and behavior in response to different stimuli, by using different materials together or alone. The method towards a broad application of CLC to be applied to future “smart” 4D structurally colored devices.

**FIGURE 7:**
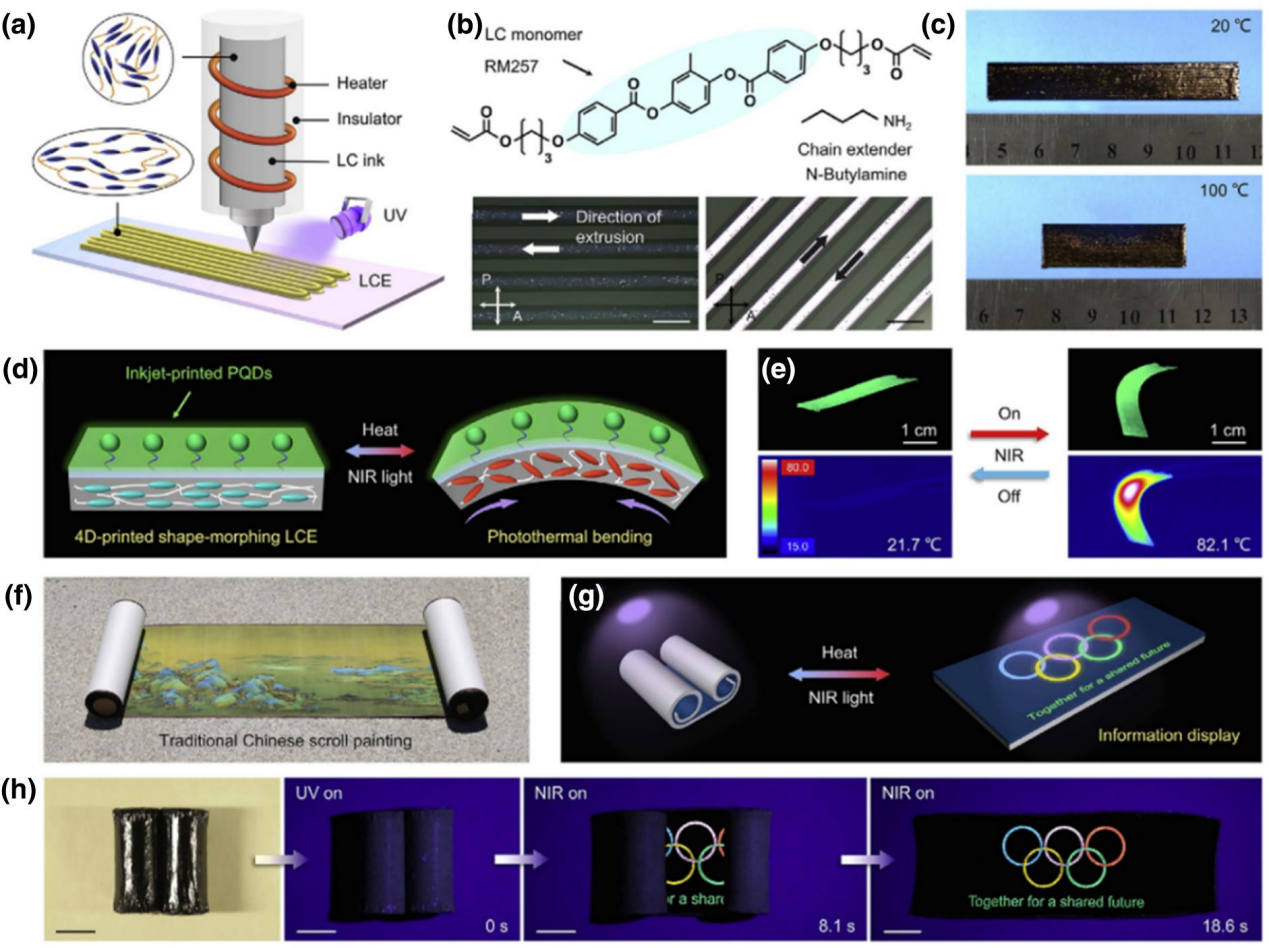
(a) Diagram shows the steps involved in making the 4D‐printed LCE film. (b) Chemical compositions of the LC monomer and the chain extender (top) and polarized optical microscopy (POM) pictures of the LCE films at 0° (dark) and 45° (bright) to the polarizer (bottom). (c) Photographs showing the contraction of the LCE film when heated from 20°C to 100°C. (d) Diagram showing the CsPbBr_3_‐LCE film's reversible shape‐bending deformation in response to heating or NIR light exposure. (e) Pictures and matching infrared thermal pictures demonstrating the CsPbBr_3_‐LCE film's shape‐bending deformation in response to UV and NIR light irradiation. (f) Image of an antique Chinese scroll painting. (g) Diagrammatic representation of an information display that may self‐deploy when heated or exposed to near‐infrared light. (h) Images demonstrating the self‐deployable method in UV and NIR light conditions.^[101]^ Copyright 2023, Elsevier Inc.

Luminescence from zero‐dimensional nanostructures (quantum dots) is one of the highlights in physics where diverse color emissions arise under various wavelength excitations. However, the combination of stretchable LCEs and luminescent materials remains a research challenge. Wang et al. proposed a method to create self‐deployable information displays by mixing 4D‐printed shape‐morphing LCEs with inkjet‐printed fluorescent perovskite quantum dots (PQDs), which are strongly covalently bonded at the organic‐inorganic interface through siloxane chemistry.^[101]^ Figure 7a shows the DIW process by which 4D‐printed LCEs are fabricated while designing for the DIW fabrication of the 4D‐printed LCE, and Figure 7b depicts the chemical structures of the chain extender and the LC monomer. When heated from room temperature to 100°C across its T_NI_, the LCE film contracted along the printing direction due to the thermally induced decrease in LC ordering, resulting in a reversible contraction of 43% (Figure 7c). As schematically illustrated in Figure 7d,e inkjet‐printed multicolored PQDs are covalently adhered to a polydimethylsiloxane (PDMS)‐LCE film so that the resulting flat CsPbBr_3_‐LCE film exhibits bright green fluorescence when exposed to UV light. When heated or exposed to NIR light, it can undergo reversible shape‐bending deformation toward the LCE side owing to its thermally or photothermally induced contraction and the PDMS passive layer. As shown in Figure 7f,g, the 4D‐printed PQD‐LCE film was designed to self‐roll at room temperature, so constructing a light‐driven self‐deployable information display that under UV light and NIR light irradiation emits fluorescent patterns (Figure 7h). This demonstration can stimulate the development of flexible functional systems and integrated devices by using a broad range of responsive polymers. Furthermore, a variety of actuating techniques, including electric and magnetic fields, could offer more prospects for polymer smart materials and their developing uses in next‐generation advanced optoelectronics and photonics.

3D printing outperforms in material flexibility, feasibility for multimaterial printing, and high resolution. Solutions, pastes, and gels of different viscosities can all be loaded into ink barrels; thus, 3D printing has been intensely involved in CLCEs‐based printing with different crosslinking degrees. This printing method is also laboratory‐friendly, especially when printing nanocomposites with different contents of nanofillers or nanoparticles, regardless of whether they are transparent or not. Moreover, the required printing conditions and setups for 3D printing are relatively simple and cost‐effective.

## 5 CHOLESTERIC LIQUID CRYSTAL ELASTOMERS VIA ANISOTROPIC DESWELLING

In 2001, Finkelmann and coworkers proposed the “anisotropic deswelling” strategy to prepare CLCE films with helical self‐assembly.^[102]^ They first prepared a precursor solution by centrifuging it at a high temperature for 10 h to trigger a hydrosilation reaction, followed by solvent evaporation. This method produced satisfactory results but it was tedious and not suitable for large‐scale preparations. The experimental protocol was later optimized by Lagerwall to eliminate the centrifugation step and reduce the self‐assembly time to 5–8 h, thus offering a method to create highly elastic CLCE films with a large surface area, millimeter thickness, and intense, uniform color reflection.^[103]^ In addition, the photonic films were also demonstrated to be repeatably, quickly, and continuously tunable across the entire visible spectrum, resulting in a method that is straightforward, replicable, and scaleable. This method takes advantage of the anisotropic volatility of the solvents to orient the helical nanostructure of CLCs, avoiding the use of centrifugation, orientation layers, or external magnetic or electric fields. The initial “click” polymerization reaction of acrylate and thiol groups in the precursor mixture formed a gel that adhered strongly to the substrate. As the solvent evaporated, the gel did not shrink in transverse dimension or change its surface area during drying. This limited solvent evaporation to the direction perpendicular to the substrate, thereby yielding a LC director with a flat internal orientation and a vertically oriented helical axis.

**FIGURE 8:**
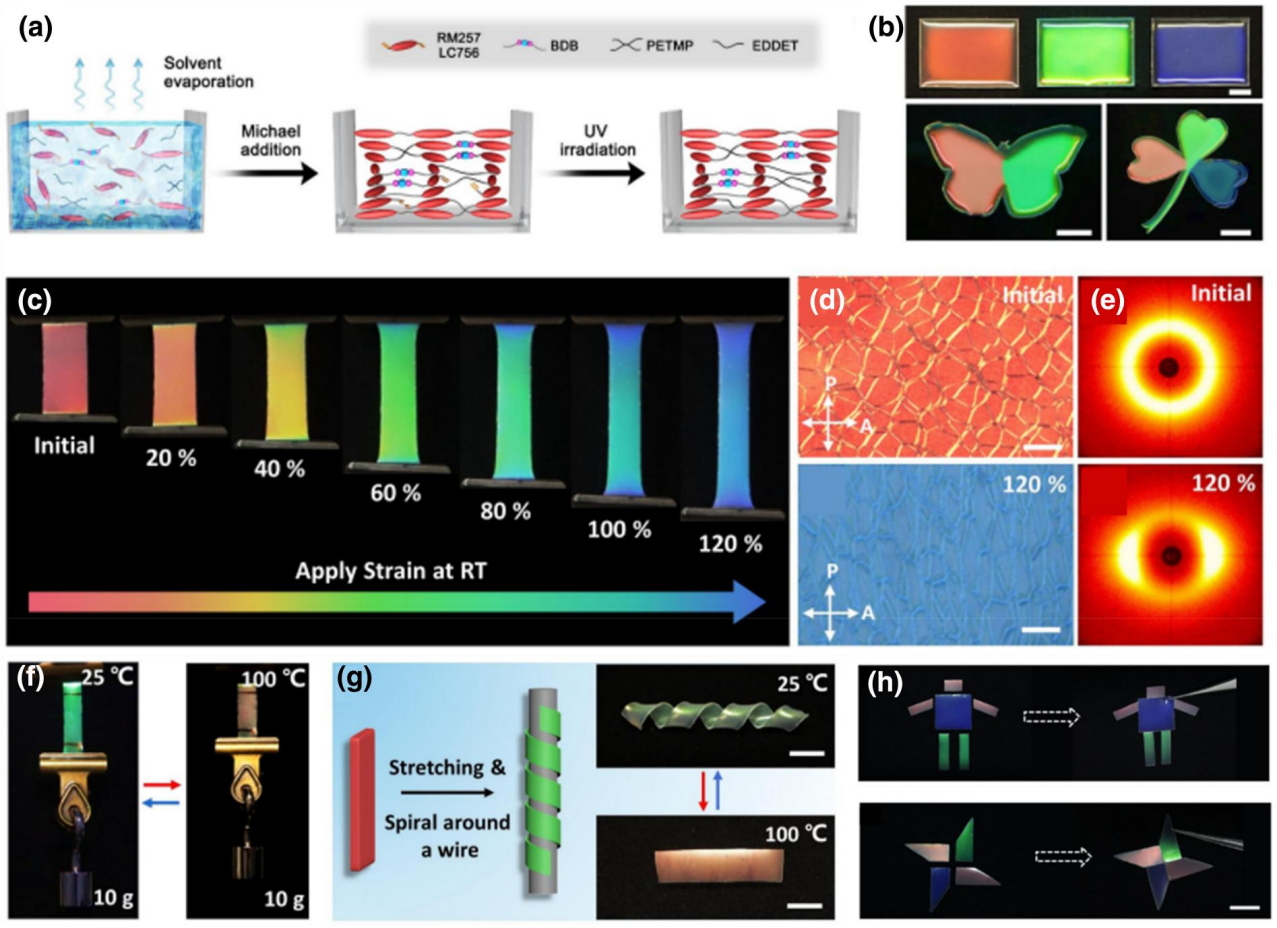
(a) Diagram illustrating the simple anisotropic deswelling method used to create CLCEs by combining photopolymerization processes with a two‐stage thiol‐acrylate Michael addition. (b) CLCE films reflect red, green, and blue colors. (c) Photographs of a red color CLCE film being mechanically stretched. (d) POM image of the unstretched polydomain CLCE and the corresponding WAXD pattern. (e) POM image of the 120% strained CLCE and the corresponding WAXD patterns exhibiting monodomain alignment. (f) Images of the programmed CLCE film lifting up a load (15 g). (g) Schematic illustration and photographs of reprogrammed CLCEs at different temperatures. (h) Visual pictures of a cartoon man design and a wind mill design assembled by the self‐healing of CLCEs.^[104]^ Copyright 2022, Wiley‐VCH.

The resulting homogeneous periodic helical laminate allows for photonic helical nanostructures within a polymer gel, giving rise to a macroscopic film with bright circularly polarized reflective colors. However, due to the previously employed chemistry, the anisotropic deswelling method produces crosslinked CLCEs with permanent covalent networks once synthesized. Wang et al. prepared CLCEs with uniform structural colors as well as shape‐programmable and self‐healable properties by combining the anisotropic volatilization method with the two‐step thiol‐acrylate Michael addition and photopolymerization reaction, and further introducing dynamic covalent boronic ester bonds into the main‐chain CLCE polymer backbone (Figure 8a).^[104]^ The CLCE precursor in toluene solution was deposited onto a glass substrate and left for 5 h at room temperature (25°C) for the first‐stage Michael addition process. Then, the sample was placed in a fume hood for another 24 h to evaporate the toluene completely. Finally, the CLCE was exposed to UV light to start the second step of photoinitiated crosslinking, as shown in Figure 8b. Interestingly, CLCEs exhibited interesting dynamic mechanochromic behaviors at room temperature. For example, mechanical stretching changed their color from red to blue continuously (Figure 8c). The mechanochromic process of the stretched CLCE film was investigated by wide‐angle X‐ray diffraction (WAXD) and POM. Figure 8d,e shows the transition of WAXD patterns from a ring to a pair of arcs along the transverse direction and the POM textures of uniaxially stretched CLCE, revealing the monodomain structure of the stretched blue‐reflecting CLCE and the polydomain structure in the initial red‐reflecting CLCE. The authors exploited the thermally initiated B‐O bond exchange property to stabilize the strain‐induced LC molecular arrangement, thus enabling us to obtain programmable CLCEs that could change shape and color reversibly when the temperature varied between 25°C and 100°C, as shown in Figure 8f,g. Moreover, breaking and reforming of dynamic B‐O bonds in water facilitated the self‐healing of colored CLCE cut pieces by adding a few drops at the broken interface to bind them close together at room temperature for 24 h (Figure 8h). This research presents a simple method for fabricating photonic CLCEs that exhibit a wide range of mechanochromic responses, 4D (color and 3D shape) programmability, and highly efficient self‐healing properties. Future “smart” soft matter technologies, such as adaptive optics, bioinspired camouflage, and somatosensory soft robots, could be made possible by further exploiting these properties.

**FIGURE 9:**
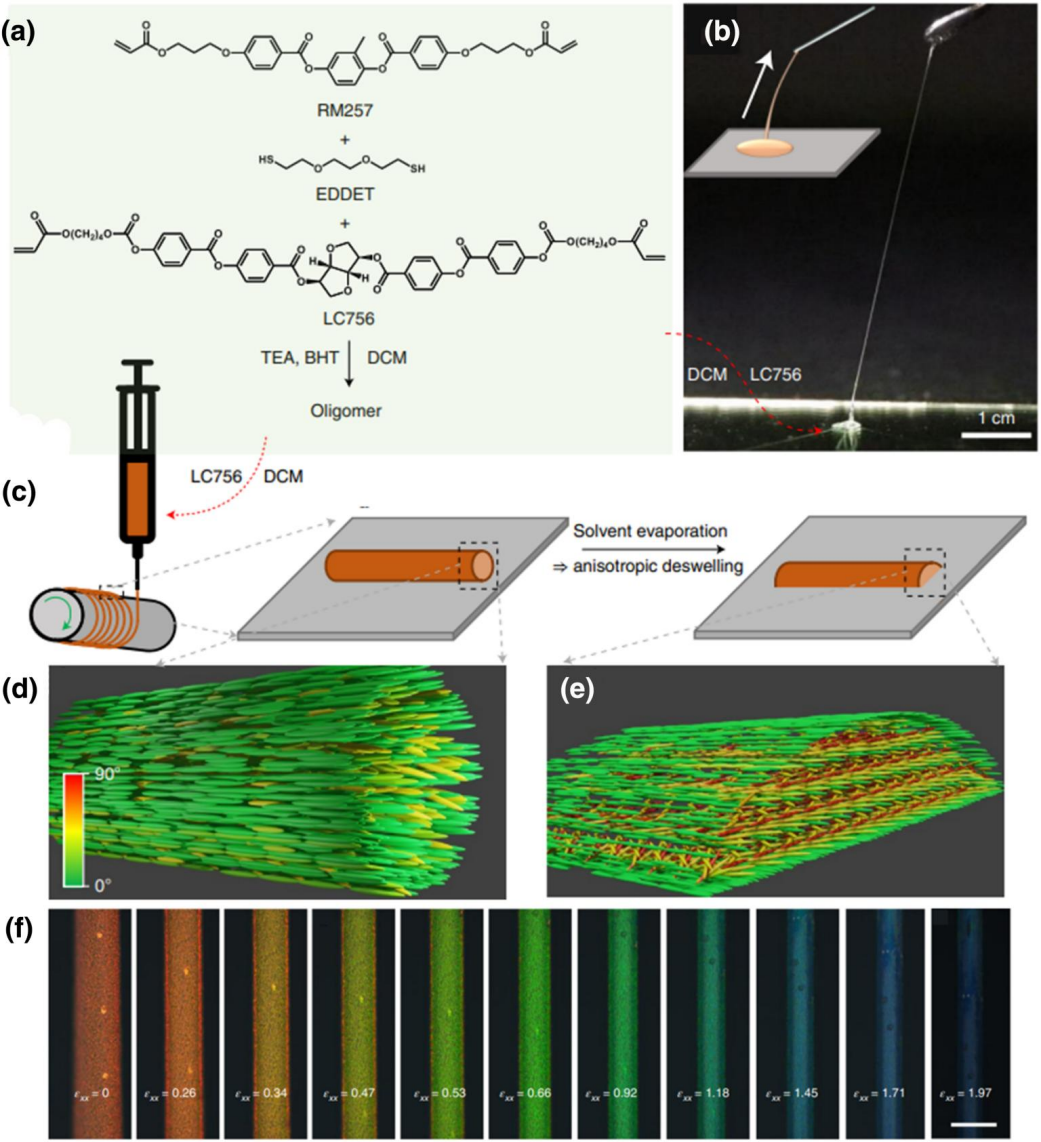
(a) Composition and synthesis of the CLC oligomer. (b) A needle can be used to extract filaments from an oligomer solution droplet. (c) Diagrams showing the filament form immediately after deposition and following anisotropic deswelling, as well as the filament extraction from a syringe carrying the oligomer solution onto the rotating mandrel. (d, e) Idealized schematics illustrating the low‐aligned paranematic (d) and the ideal cholesteric structure with vertical helix promoted by anisotropic deswelling (e). (f) POM pictures in reflection mode of a CLCE fiber during elongational strain that initially reflected red light.^[52]^ Copyright 2022, Springer Nature.

Structural color applied to textiles has attracted increasing attention worldwide,^[105]^ but has mostly been developed using building block nanoparticles which limit the response range and elasticity. Fibers, as the basic unit of fabrics, can determine the fundamental characteristics of textile products, so they are considered the basis for conferring stimulus‐response to photonic fabrics. However, constructing mechanochromic CLCE fibers is difficult because the Plateau‐Rayleigh instability breaks precursor solutions into droplets, which prevents continuous filament extraction. Lagerwall et al. reported the fabrication of CLCE fibers from an acrylate‐terminated oligomeric precursor synthesized by thiol‐acrylate Michael addition polymerization (Figure 9a), which was later gently diluted and subsequently extracted to form photonic filaments benefiting from the anisotropic deswelling method.^[52]^ They balanced the viscoelastic characteristics to allow continuous filament extraction and delay the Plateau‐Rayleigh instability until the helix structure was self‐assembled (Figure 9b). Figure 9c schematically depicts a straightforward setup to generate long fibers with well‐controlled dimensions and mechanochromic properties in a reproducible way. The filament diameter could be varied from micrometers to millimeters by adjusting the set feed rate Q, speed v, and rotation speed *ω*. As shown in Figure 9c, the filament had a circular cylindrical cross‐section when drawn from the syringe into the air, but quickly deformed into a hemicylindrical shape as soon as it was deposited on the mandrel. The precursor exhibited an isotropic phase at rest, but the extensional flow aligned the oligomer uniaxially, resulting in a paranematic state along the filament director, as shown in Figure 9d. The filament was then relaxed and photopolymerized by UV irradiation to produce the final CLCE fiber. After about 10 h of vertical helix formation (Figure 9e), the optical properties changed as the solvent evaporated, and the first clear sign of colored selective reflection emerged. The relaxed red retroreflective fiber gave the optimal mechanochromic response through the entire visible spectrum when subjected to high elongational strain (Figure 9f). The fibers could also be integrated into garments demonstrating that they are durable enough to endure frequent stretching and machine washing. This alignment approach and the photonic fibers could benefit applications in wearable technology and other fields that require self‐powered strain sensing or monitoring of extreme deformations.

**FIGURE 10:**
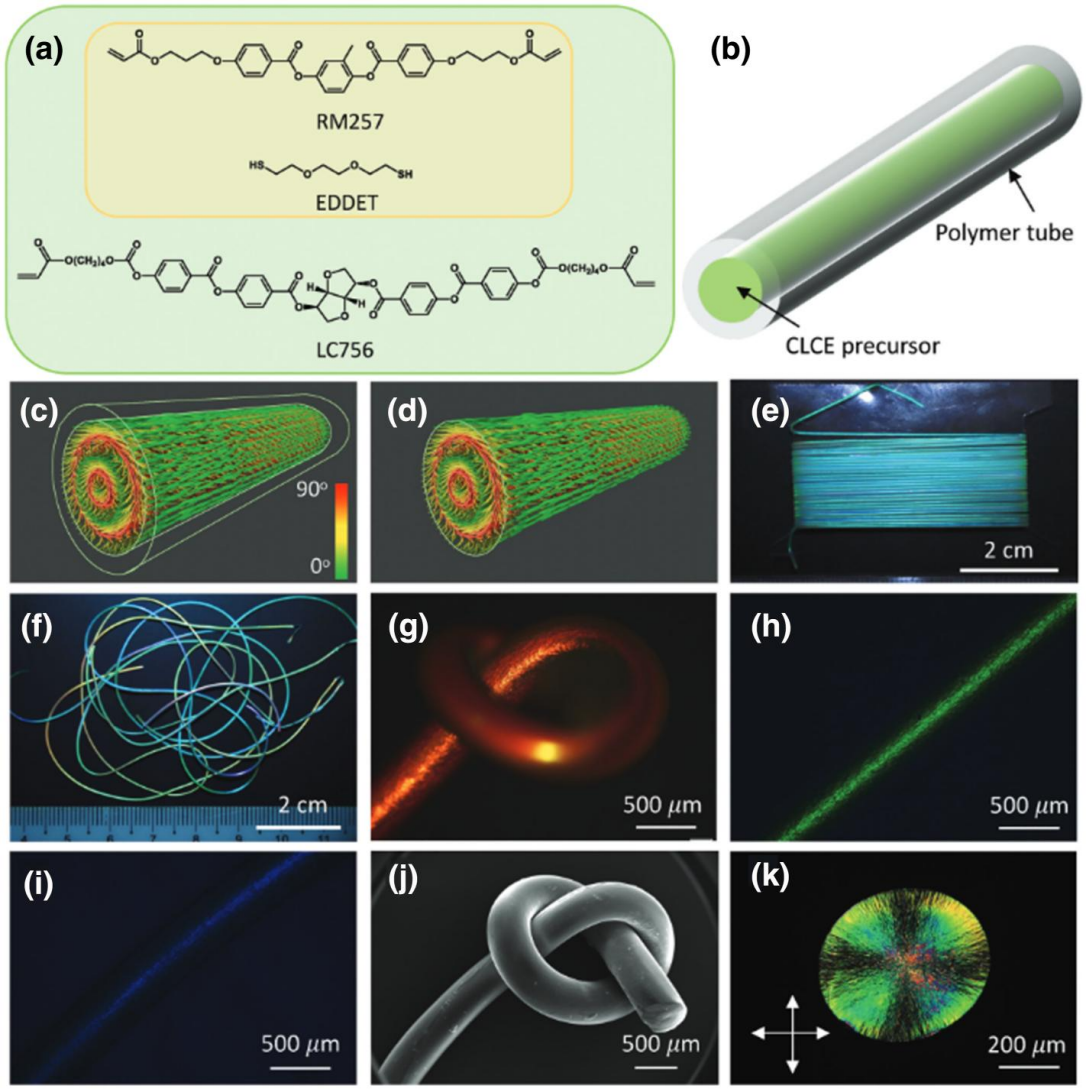
(a) Before tube filling, chiral dopant LC756 is injected to convert the phase cholesteric. Reactive LC monomer RM257 and flexible chain extender EDDET react to generate nematic oligomers. (b) The CLCE precursor is introduced into an inner diameter 0.5–0.6 mm LDPE tube. (c) Schematic drawings of the cholesteric order with radial helix developing within the tube; and (d) after crosslinking and tube removal. (e, f) Macroscopic views of (e) 2.5 m of CLCE fiber with blue‐green λ∗_0_ collected on a flat winder, and (f) of several fibers with red, green and blue λ∗_0_, respectively. (g–i) Images of CLCE fibers with reflection POM (crossed polarizers) and red, green, and blue λ∗_0_, respectively. (j) SEM image of a red λ∗_0_ CLCE fiber knot is seen in the reflection POM in (g). (k) An image of a 5 μm thick CLCE fiber cross‐section slice captured using transmission POM (crossed polarizers).^[106]^ Copyright 2023, Wiley‐VCH.

The continuous extrusion and deposition of the oligomeric CLCE precursor solution on a rotating mandrel, however, causes the process to yield belt‐shaped fibers that are far from ideal cylindrical symmetrical CLCE fibers, but most of all, it makes the process very sensitive to failure, which constitutes a major obstacle to the scale‐up of this method radial orientation of the cholesteric helix. In order to address these drawbacks, Lagerwall et al. reported cylindrically symmetric CLCE fibers annealed within a sacrificial tube to radially orient the cholesteric helix, which exhibited enhanced thermochromic, thermomechanical, and mechanochromic responses.^[106]^ Typical thiol‐acrylate Michael addition reaction in dichloromethane resulted in an oligomer precursor that was subsequently mixed with the chiral dopant and capillary introduced into a low‐density polyethylene (LDPE) tube (Figure 10a,b). Since LDPE is permeable dichloromethane, diffusion and consequently continuous evaporation of the solvent through the tube wall for 2 days at 45°C allowed the CLC phase to self‐assemble with a radially oriented helical pitch (Figure 10c). When the cylindrically symmetric cholesteric order was stable, the precursor was crosslinked by UV irradiation through the tube wall and submerged in toluene for 30 min in a closed vessel at 90–100°C to dissolve the LDPE tube. Figure 10d schematically illustrates the radial helix alignment fixed by the polymer network, and experimentally confirmed by the birefringent fingerprint of the Maltese cross (Figure 10k), whereas Figure 10e shows a 2.5 m long CLCE fiber fabricated by this method. The ground state retroreflection wavelength (λ∗_0_) of different fibers could be easily controlled over the visible spectrum or into the near‐infrared (IR) spectrum by altering the concentration of the chiral dopant, as shown in Figure 10f. Furthermore, high color contrast against any background is achieved for the CLCE fiber (Figure 10g–i), which is derived from the production process that guarantees that the fibers adopt a cylindrical shape with uniform smooth surface, diameter, and identical responses in all directions perpendicular to the fiber axis, as seen in the scanning electron microscopy images in Figure 10j.

Beyond uniaxial deformation, Choi et al. studied how multiaxial (uniaxial, biaxial, and out‐of‐plane) stretching affects the optical properties of CLCEs by inducing helical structural changes.^[107]^ The results show that biaxial and out‐of‐plane stretching preserve circular polarization better than uniaxial stretching. This is because the former deformations expand in two perpendicular directions, while the latter deformation unwinds the helix and destroys its circular polarization.

**FIGURE 11:**
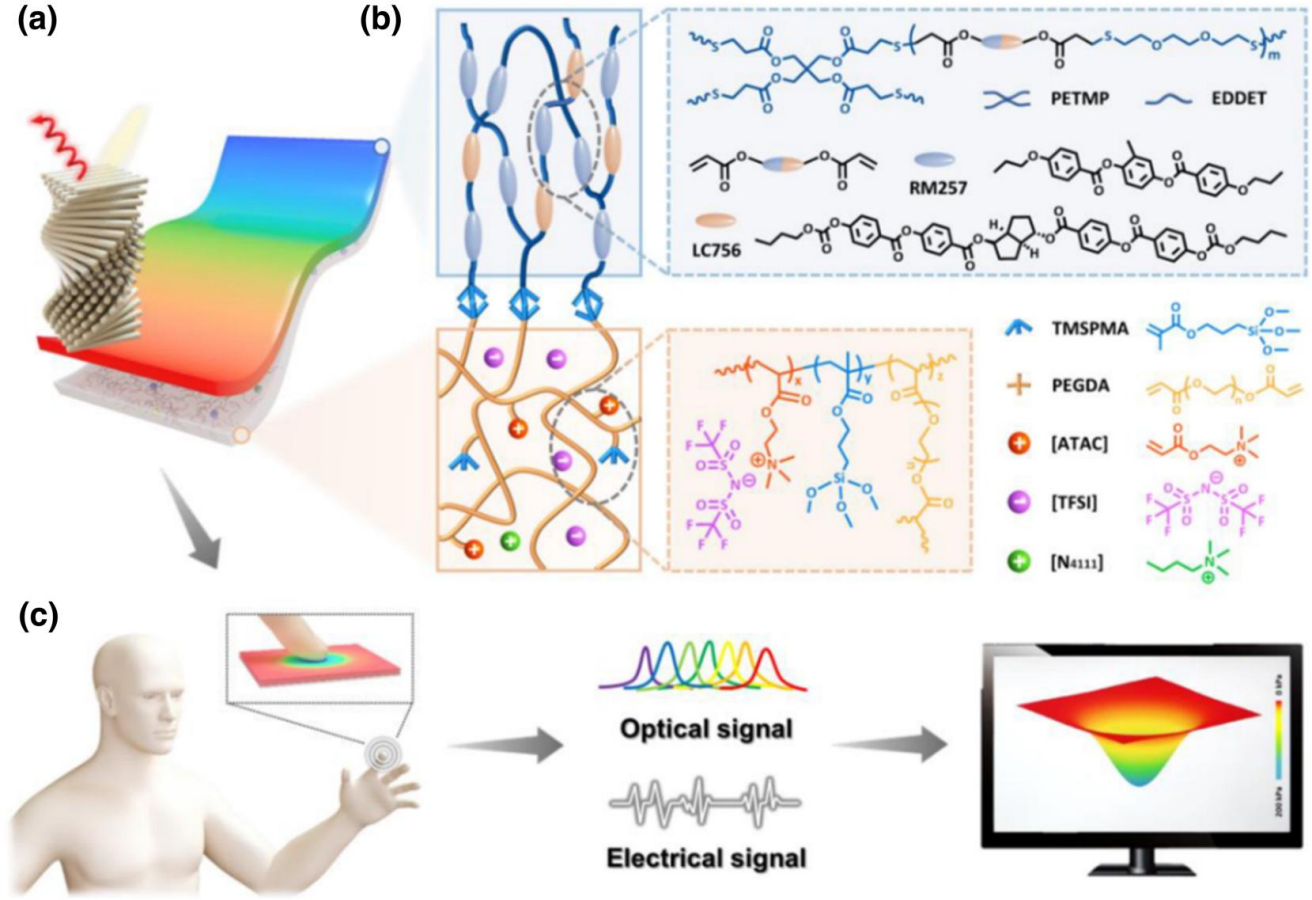
Design and concept of the iCLCEs. (a) Diagrammatic representation of the iCLCEs. (b) The silane coupling layer at the interface, PILNs, and CLCE make up the chemical compositions of the iCLCEs. (c) Performance of the iCLCEs in dual‐signal sensing (optical and electrical).^[108]^ Copyright 2023, RSC.

**FIGURE 12:**
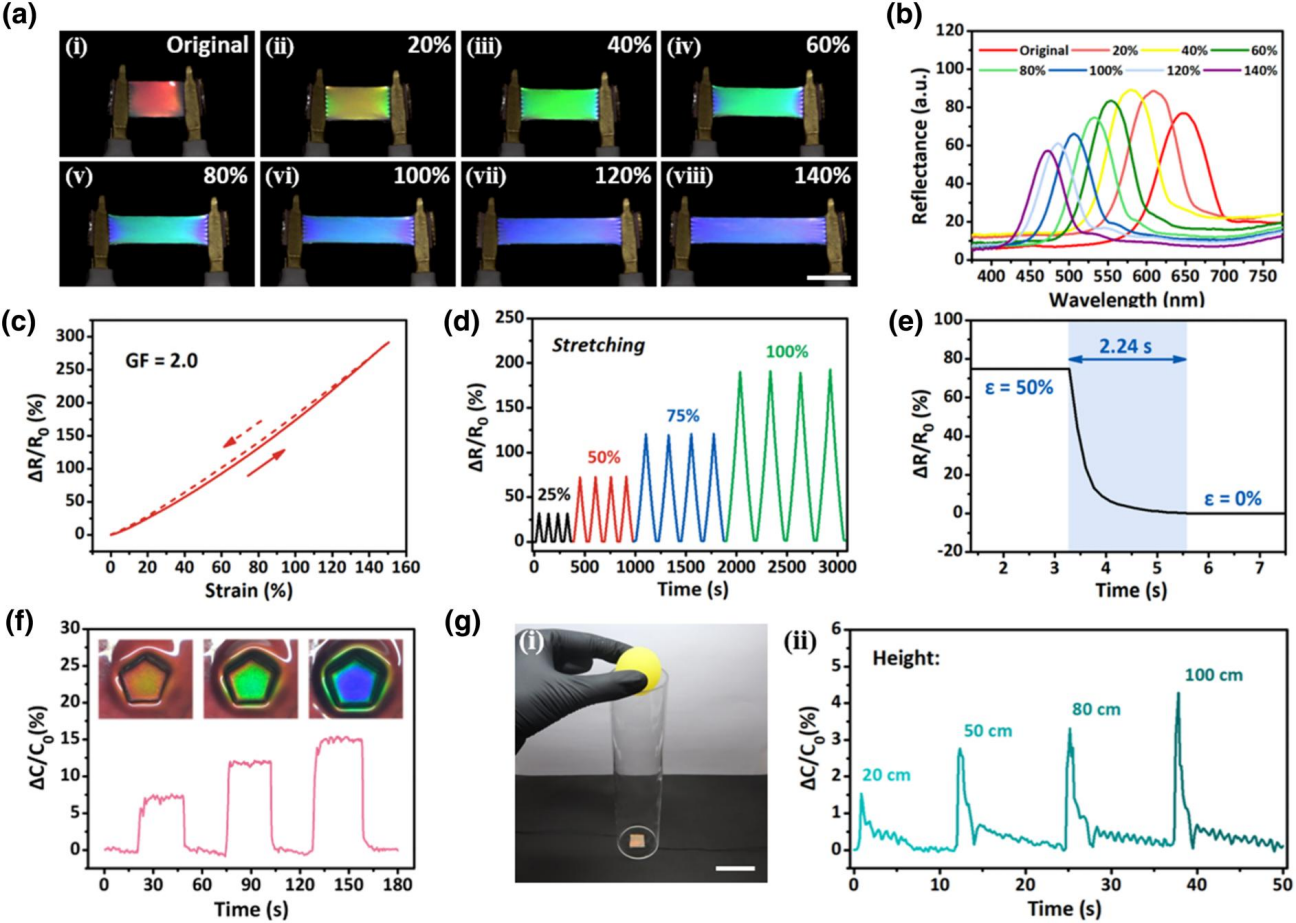
Optical, electrical and capacitive sensor properties of iCLCEs. Images and corresponding reflection spectra of the red‐reflecting iCLCE that is mechanically stretched from 0% to 140% are shown in (a) and (b). (c) Changes in iCLCE resistance that depend on strain. (d) Relative resistance varies between different strains. (e) Relative resistance signal's self‐recovery time from 50% to 0%. (g) Relative capacitance reactions to a pressure source in the pentagon form. Corresponding structural color changes are displayed in the insets. (h) The schematic configuration (i) and the capacitive sensor's real‐time signal response (ii) to a bouncing ball falling from various heights are shown.^[108]^ Copyright 2023, RSC.

Despite the extensive efforts to create and improve the color‐changing ability of CLCEs, conductive CLCEs with sensing capabilities have not yet been demonstrated. Wang et al. designed and fabricated ionic conductive (iCLCEs) on silane‐functionalized polymer ionic liquid networks using free‐radical photopolymerization of the CLCE precursor and in situ Michael addition (Figure 11).^[108]^ The iCLCEs have tuneable helical nanostructures that enable them to show remarkable dynamic mechanochromic behaviors at room temperature. When stretched from the initial state to 140% strain, the iCLCE exhibits sensitive and reversible color switching across the full spectrum from red to green to blue, as shown in Figure 12a. The reflection wavelength shifts continuously from 647 to 471 nm, resulting in mechanochromic sensitivity of 1.26 nm %^−1^ (Δ*λ*/Δ*ε*, where *λ* is the reflection wavelength and *ε* is the strain), as shown in Figure 12b. The relative resistance increases gradually with increasing strain (Δ*R*/*R*_0_ = (*R* − *R*_0_)/*R*_0_, where *R*_0_ is the initial resistance and *R* is the test resistance). The gauge factor is 2.0 (GF = (Δ*R*/*R*_0_)/ε), implying good sensitivity to detect the mechanical strain (Figure 12c). The iCLCEs show repeatable and strain‐dependent resistance changes under fixed strains of 25%, 50%, 75%, and 100%, as shown in Figure 12d. This shows that the system is capable of identifying various amplitudes of mechanical deformation. The response time of the iCLCEs shown in the self‐recovery settings is displayed in Figure 12e. The capacitive sensor was loaded and unloaded more than 200 times under the same pressure in order to assess its durability. As seen in Figure 12f, the extremely steady capacitance signals support the superior pressure sensing capability. Figure 12g shows a proof‐of‐concept illustration of applying a pentagon‐shaped pressure source to the capacitive sensor. This strategy can provide new insights for designing and fabricating bioinspired multifunctional photonic materials that can be used for a human‐machine interaction, visualized interactive devices, and advanced artificial skins.

CLCEs based on the anisotropic deswelling can be easily prepared into CLCE fibers and other different patterns, and have a wide range of applications in wearable sensing, information anti‐counterfeiting and other fields. In addition, CLCE prepared using various anisotropic deswelling has a good match with organic and inorganic materials, and can prepare composite devices with multiple functions, which shows excellent application prospects in human‐computer interaction, intelligent robots, etc. However, if this type of composite device lacks chemical bonds to achieve interface assembly, its tensile stability and repeatability will be poor, making it difficult to implement it in related applications.

## 6 BLUE PHASE LIQUID CRYSTALS WITH 3D NANOSTRUCTURE

The blue phase (BP) is a type of LC phase with cubic lattice structures that occurs between the isotropic and cholesteric phases. The BPLCs possess unique optical properties, such as optical anisotropy, fast electric field response, and selective reflective wavelengths, which render them promising materials for several fields, including LC displays, optical devices, and tunable 3D photonic crystals.^[109]^ In addition, the natural self‐assembly of highly ordered 3D nanostructures in BPLCs endows soft photonic crystals with an unparalleled capacity to control the flow of photons in three dimensions, as well as eliminating the need for tedious molecular alignment that is otherwise performed using conventional LCs.^[3]^ To form 3D lattices, the CLC monomer is injected into a cartridge and heated up to its isotropic temperature. Then, the material is slowly cooled down within a temperature range to allow the self‐assembly of the crystals. Depending on their chirality after cooling from the isotropic phase to the cholesteric phase, BPs can be categorized into three types: BP I, BP II, and BP III. BP I and BP II have body‐centered cubic nanostructures and simple cubic symmetry, respectively. BP III has a disordered amorphous nanostructure.^[56]^ Although defects or disclinations in BP nanostructures are inevitable since the double‐twisted cylinders cannot constantly fill the entire 3D space, the main driving force behind these studies opens the door for bestowing fast electro‐optical switching properties in systems that do not require precise thickness control or alignment layers.

On‐demand controllable optical qualities imply an effective response to environmental changes and external stimuli, among which the electric field, humidity, and ambient temperature become more salient for BPLCs as those factors can alter their BP lattice orientation and transform their BP nanostructures. For example, Yang and coworkers reported the fabrication of BPLC‐based photonic shape‐memory polymers capable of exhibiting high visible‐wavelength reflectivity and low photonic band gaps.^[110]^ By using a shape memory programming procedure, the free‐standing blue phase films displayed various blue‐shift colors under different mechanical pressures. The distorted BP films could be restored to their original shapes and reflecting colors when heated above the glass transition temperature. Responsive BPLC polymer coatings with 3D photonic nanostructures and novel functionalities have also been published by Yang et al., who developed humidity‐driven color‐changing photonic polymer coatings based on carefully designed hydrogen‐bonded 3D BPLC networks.^[61]^ These findings are expected to provide new insights into the creation of sophisticated functional materials that respond to stimuli and have flexible 3D photonic nanostructures for various technological applications, such as anticounterfeiting, display, biomimetic camouflage, and sensing.

**FIGURE 13:**
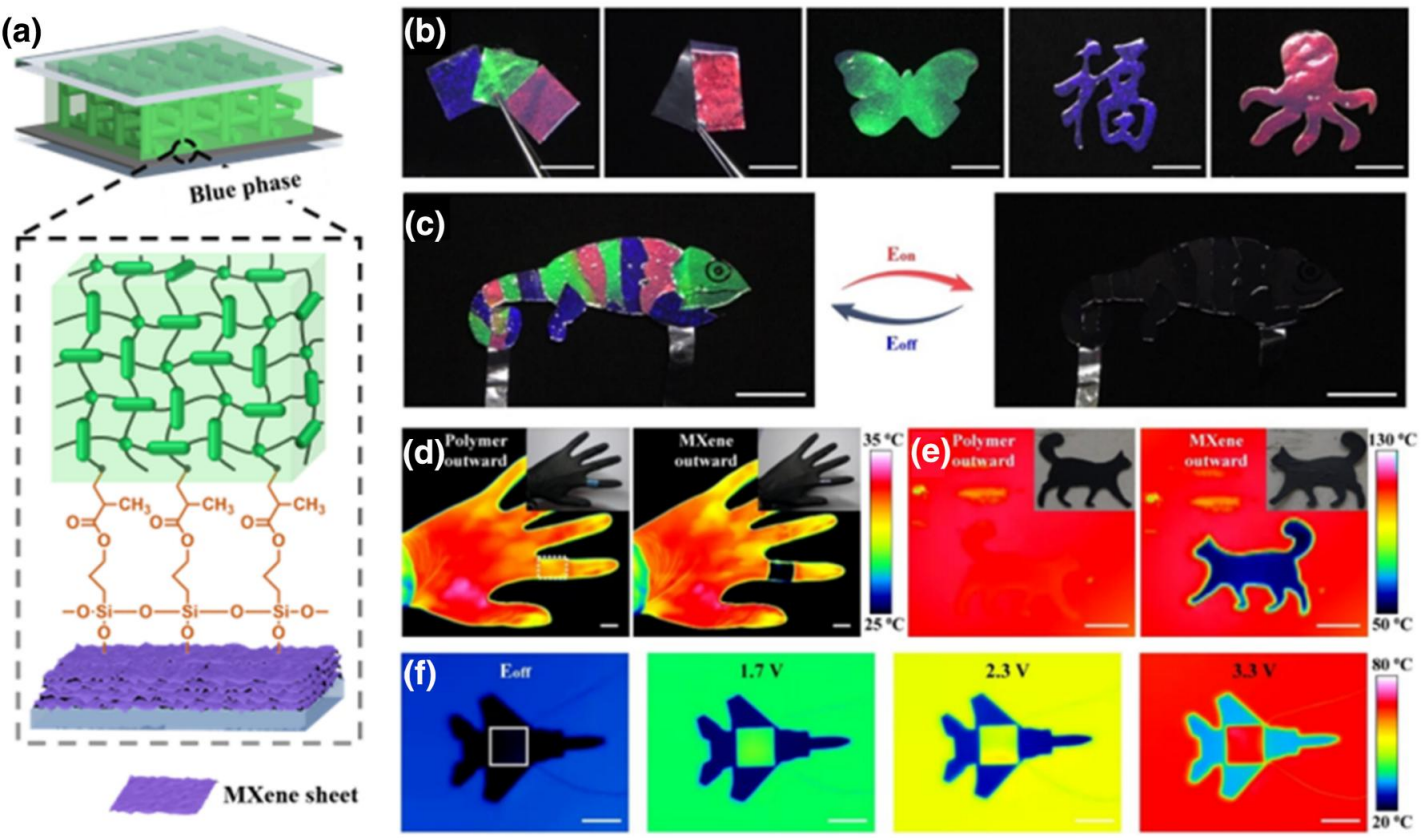
(a) Chemically attaching BPLCs to a functional silane‐treated MXene thin film creates a strong covalent chemical bond. (b) Electrochromic films created by laser cutting with various colorful designs. (c) Integrated colorful electrochromic flexible films with an artificial chameleon. Flexible films' Janus thermal camouflage characteristic at (d) room temperature and (e) high temperature. (f) Flexible film's dynamic thermal camouflage under voltage variations^[111]^ Copyright 2022, Wiley‐VCH.

Wang et al. proposed an emerging method for synthesizing dynamic visible and IR camouflage materials by carefully controlling the in situ fabrication of novel photopolymerizable BPLCs with cubic nanoarchitectures on perfectly aligned MXene nanostructured thin films.^[111]^ 3‐(trimethoxysilyl)propyl methacrylate (TMSPMA) anchoring layer created strong covalent bonds between the interfacial surface of MXene and BPLCs, resulting in MXene‐integrated 3D soft photonic crystals (Figure 13a). The resulting 3D soft photonic crystals have brilliant structural colors and can switch between a brightly colored state and a black state under a weak DC electric field. Free‐standing electrochromic flexible films with various reflection colors were also produced using MXene‐integrated 3D soft photonic crystals over a layer of optically transparent PVA polymer. In the experiment, free‐standing electrochromic flexible films with various reflection colors could be easily created into various colorful patterns, such as the “Fu” character, an octopus, and a butterfly (Figure 13b). As an example of proof‐of‐concept, a synthetic chameleon was created by combining electrochromic flexible films with various colors, which can switch between a bright‐colored state and a black state by applying a low DC voltage (Figure 13c). Moreover, the free‐standing electrochromic flexible sheets have outstanding Janus thermal camouflage properties, as shown by a thermal imaging camera. Figure 13d shows the visual and infrared pictures of a green flexible film uniformly wrapped around the index finger, and the thermal camouflage feature even persists at high temperatures due to phase transition when heated up to 110°C with the polymer layer facing outward, making it invisible to a thermal imaging camera (Figure 13e). Figure 11f shows an airplane model coated with a square (1 × 1 cm^2^) electrochromic flexible MXene‐integrated 3D photonic sheet, which was connected to a DC power supply through the MXene layer. The contrast of the IR pictures makes it easy to recognize an airplane model at low temperatures. By applying appropriate voltages of different magnitudes, the temperature of the electrochromic flexible film can be adjusted to match the ambient thermal radiation. The region coated with the electrochromic flexible film can thus be easily hidden in the surroundings. This research could lead to the development of sophisticated functional nanoarchitectures and intelligent adaptive materials for various novel emergent applications, such as bioinspired camouflage, advanced temperature control, wearable electronics, programmable optoelectronics, and beyond.

It is important to note that the aforementioned works unveiled BPLCs with poor stretch properties often retained in glassy polymers upon cross‐linking, while it is well known that BPLCEs could provide great potential for applications ranging from strain sensing to soft wearable electronics. In 2014, using a stretchable gel of BP I, Coles et al. reported the construction of a self‐assembling, 3D photonic crystal that is electro‐optically switchable under a moderately applied voltage and whose optical characteristics are controllable by applied strain.^[112]^ Additionally, authors discovered that, in contrast to its undistorted counterpart, a mechanically deformed blue phase shows a Pockels electro‐optic effect, which would open up new theoretical opportunities and problems for low‐voltage electro‐optic devices.

**FIGURE 14:**
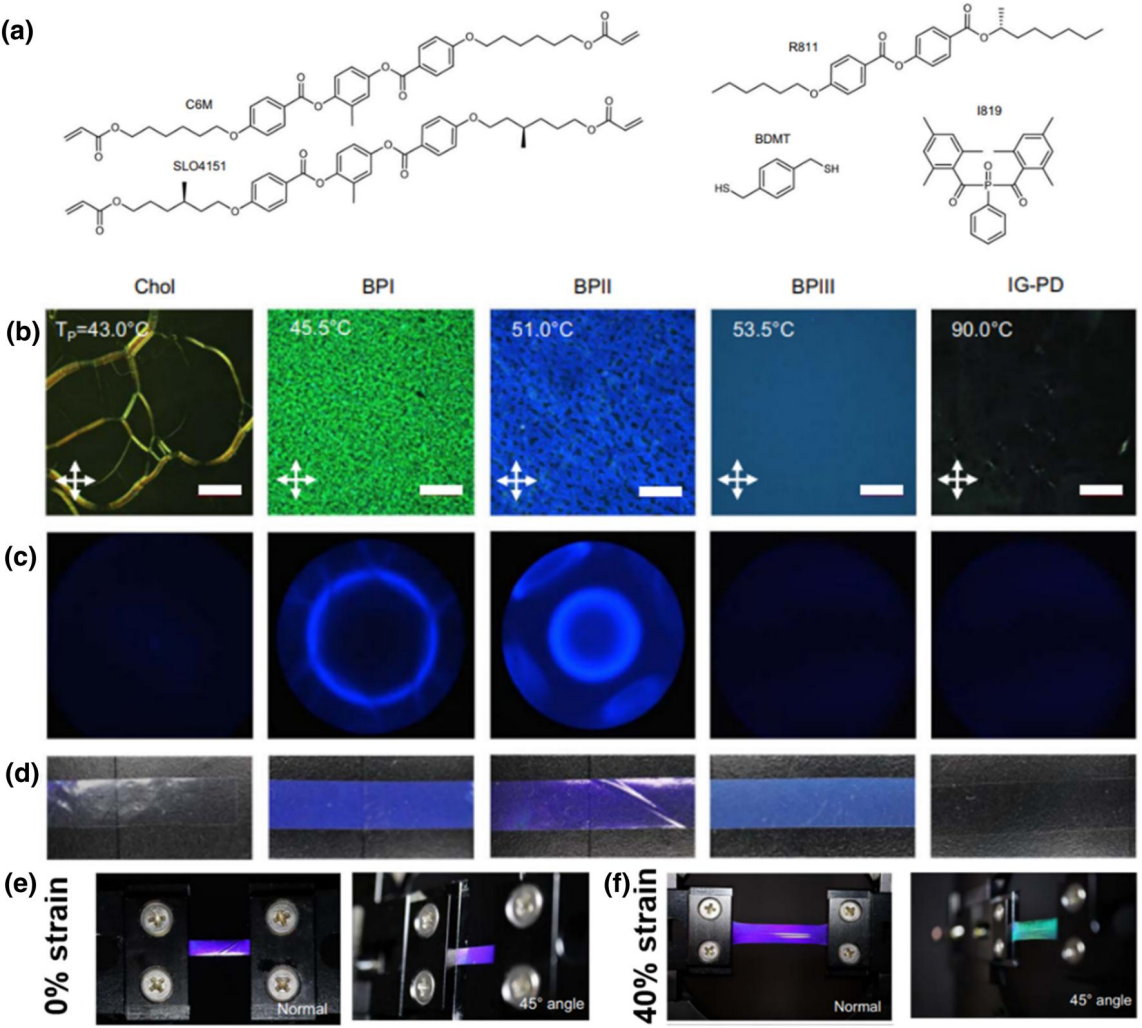
(a) Chemical structures of monomers. (b) Textures of the LCE's POM in reflection mode. Following each phase's retention by photopolymerization at the polymerization temperature (TP), images are taken at room temperature. The blue phases are blue phase I (BPI), blue phase II (BPII), blue phase III (BPIII), cholesteric phase (Chol), and isotropic genesis polydomain nematic (IG‐PD). (c) Kossel diagram (440 nm) of free‐standing elastomers that maintain the stated phase and verify the cubic structures for BPII (simple cubic [100]) and blue phase I (body‐centered cubic [110]). (d) Images of LCE that show either IG‐PD phase, unpolarized illumination or any combination of Chol, BPI, BPII, BPIII, or BPI. (e) Images of unrestrained BPII LCE with light coming from the side of the camera. (f) Images of a 40% strained BPII LCE that were lit from the front of the camera.^[113]^ Copyright 2021, Springer Nature.

Despite the fact that polymer‐stabilized BP gels were demonstrated to display strain‐induced color changes over almost the entire region of visible wavelength, high photonic sensitivity to mechanical deformation and electro‐optical responses in highly stretchable elastomers with glass transition temperatures below room temperature remain a challenge. Schlafmann et al. created a fully solid BPLCE with dynamically reconfigurable 3D nanostructures after photopolymerization.^[113]^ The composition (Figure 14a) consists of CLC diacrylate SLO4151 and C6M, with nonreactive chiral dopant R811 (15%), benzenedimethanethiol (BDMT) as a chain extender and transfer agent, and Omnirad819 as a photoinitiator. The photonic LCEs retain the distinctive birefringent patterns of three types of BPs after polymerization, as shown by POM images in Figure 14b. Kossel diffraction measurements confirmed the formation of a 3D lattice and the cubic periodicity of LCEs (Figure 14c). The images of the free‐standing LCE films (Figure 14d) show a slight blue shift in reflection color compared to the POM photos in Figure 14b due to the removal of the nonreactive chiral dopant from the LCE network. To further describe how mechanical deformation affects the spectrum of the lattice, Figure 14e,f show the optical properties of a BPII LCE during deformation. The images in Figure 14e show that between the normal incidence 0° and 45° sample angles, the unstrained BPII LCE changes slightly from blue to purple, mainly ascribed to the reflection associated with both the [100] and [010]/[001] planes, respectively. As the BPII LCE film thickness decreases due to uniaxial deformation at 40% strain, the reflection of the [100] lattice plane at normal incidence shifts to blue, while a red‐shifted reflection of the [010]/[001] plane becomes visible at an acute angle (Figure 14f). Moreover, these free‐standing stretchable photonic films also respond distinctively to thermal and chemical stimuli. In either case, whether the strain is symmetric (by heat or swelling) or asymmetric (by mechanical force), the deformation is always reversible, which would further extend the application scope of BPLCEs to nonlinear optics, lasers, spectral imaging, and sensing. Recently, adopting the most popular protocol for LCE synthesis, that is, the thiol‐acrylate Michael addition reaction and photopolymerization, Zhu and coworkers developed homogeneous and high‐performance BPLCE films with low glass‐transition temperature (−34.93°C), high stretchability (310% strain), and long wavelength shift (>186) from red (628 nm) to green and then to blue (442 nm).^[114]^ This research provides new insights into adaptable 3D photonic nanostructured materials that are stimuli‐responsive facilitating their use for sensing, displays, and counterfeit detection. A major advantage of preparing functional materials from BPs is the ability to form single crystals over a large area and thickness. For BPLCs, single‐crystalline assemblies with a thickness of about ∼30 μm can be prepared in a few hours due to the fast dynamics in the liquid state. As a lightweight, all‐solid, deformable optical material, BPLCE has important functional uses in nonlinear optics, lasers, spectral imaging, and sensing.

## 7 CONCLUSION

In summary, we discuss recent state‐of‐the‐art advances in smart chiral liquid crystal elastomers and provide a systematic overview of the preparation of CLCEs and BPLCEs. CLCEs have a fantastic helical nanostructure that can reflect circularly polarized light of the same handedness as the CLCE helix following Bragg's law. When CLCE films are stretched, this reduces their thickness and changes their reflected color. A variety of methods have been developed to obtain CLCE samples. Among these techniques, surface‐enforced alignment has been seen as the traditional method for preparing CLCEs, which is easily accomplished to obtain a homogeneous structure. Bar‐coating is another common method for preparing CLCEs with helical orientations aided by shear forces without the need of additional alignment layer. This method can be used for large‐area applications, owing to their thickness, shape, and flexibility could be adjusted. 3D printing is an emerging method for the preparation of CLCEs, which offers a new route to producing objects of arbitrarily large size. Currently, 3D printing of CLCEs is limited to the DIW method, which results in actuators combining pigmental color and structural changes. The anisotropic deswelling method allows to easily produce a large number of CLCE films with excellent mechanical properties and a high degree of helical orientation. The mechanisms, advantages, disadvantages, application, and materials of these preparation methods suited for chiral liquid crystal elastomers are summarized in Table 1. Furthermore, the self‐assembly of BPLCs with distinct cubic lattices requires no orientation and occurs spontaneously during the cooling process. To meet the demands of practical applications, developing precise, fast‐response, and versatile preparation methods for LCEs will be a major challenge in the future.

**TABLE 1:** Comparison of different preparation methods for chiral liquid crystal elastomers.

	Mechanisms	Advantages	Disadvantages	Application	Materials	Ref.
Surface‐enforced alignment	Shear stress	Uniform structural color	Device thickness limitation	Cryptography	Low viscosity oligomer prepared with reactive mesogens chiral dopant and dithiol chain‐extender; photo crosslinker	[82, 83, 84]
	Intermolecular force and microchannel capillary force			Adaptive optics		
				Soft robotics		
				Mechano‐optical sensors		
		Spatial control of the alignment with micrometer scale resolution				
	Photoalignment					
Bar coating	Shear stress	Quickly prepared on a large area	Difficult to obtain uniform structural colors	Specialized anticounterfeit markers	Oligomer ink prepared with reactive mesogens and dithiol chain‐extender; photo crosslinker	[27, 89]
					Two‐stage thiol‐acrylate	
	Rubbing stress	Without additional layer		Multi‐color concealed camouflage switching	Michael addition and photopolymerization reactions	
3D printing	Shear stress	Rapid printing speed	Lacking smooth and flat surfaces	Multimaterial stimuli‐responsive actuator	Oligomer ink prepared with reactive mesogens and dithiol/diamine chain‐extender; photo crosslinker	[103, 104]
			Printed structures tend to show slight distortion	Information display		
		Materials flexibility		Interactive soft multiactuator		
		Complex shape changes				
		High throughput				
Anisotropic deswelling	Compressive stress	Uniform structural color	The time for volatile self‐assembly is relatively long	Human‐machine interaction	Two‐stage thiol‐acrylate Michael addition and photopolymerization reactions	[52, 106, 107, 110]
		Large area preparation		Prostheses intelligent robots		
		Excellent mechanical performance				
				Wearable technology		
	Shear stress					

As more chemistry and alignment techniques for chiral liquid crystal elastomers are developed, we foresee that the applications of chiral liquid crystal elastomers will become more diversified. There are many opportunities and challenges in accelerating the maturation of this exciting field. The salient properties of smart chiral liquid crystal elastomers beyond reversible color change, such as soft elasticity, will begin to be exploited by engineers. Recent developments could make it possible to employ new methods when creating substrates for stretchable electronics.^[108]^ Researchers have also begun to explore other preparation strategies to enrich the functions and applications of chiral liquid crystal elastomers, such as using spinning technology to produce CLCE fibers,^[106]^ fabricating CLC droplets via glass‐capillary‐based microfluidic technologies,[^115^, ^116^] or preparing composite materials by doping gold nanorods, ionic liquid, graphene, liquid metals, and MXene to give them more functional properties.[^117^, ^118^, ^119^, ^120^] Moreover, many achiral or chiral luminescent materials have been widely used as emitters to construct CPL‐active materials based on chiral liquid crystal elastomers, such as organic fluorescence molecules, luminogens with aggregation‐induced emission (AIE) property,^[121]^ carbon dots (CDots),^[122]^ quantum rods (QRs),^[123]^ conjugated polymers,^[124]^ semiconductor quantum nanocrystals,^[125]^ and inorganic perovskite nanocrystals (PKNCs).^[126]^ The use of different types of emitters has also greatly enriched the CPL‐active materials based on chiral liquid crystal elastomers, endowing the resulting system with diverse properties.^[126]^ Li et al. fabricated LCEs that can change their shape and fluorescence color in response to light, mimicking the biological functions of some organisms such as caterpillars and frillneck lizards.^[127]^ Guo et al. reported circularly polarized room temperature phosphorescence (CPRTP) with high and switchable luminescence dissymmetric factors by using CLCEs as hosts for a phosphorescent guest molecule.^[128]^ In addition, the fascinating properties of smart chiral liquid crystal elastomer‐based CPL systems can be used to construct smart photonic materials for 3D display, sensing, and encryption applications.[^129^, ^130^, ^131^, ^132^, ^133^] Smart chiral liquid crystal elastomers could lead to functional and intelligent materials with high adaptability and potential. With the development of material systems and preparation technologies, the study of smart chiral liquid crystal elastomers will provide new ideas for the development of bionics, kinetic photonics, intelligent soft robots, and wearable devices.

## CONFLICT OF INTEREST STATEMENT

There are no conflicts to declare.

## Supporting information

Supporting Information S1

## Data Availability

The data that support the findings of this study are openly available.
