# Supplementary material for: Smart chiral liquid crystal elastomers: Design, properties and application
Source: Smart Mol. 2024 Feb 29;2(1):e20230025. doi: 10.1002/smo.20230025 (PMC12118170; doi:10.1002/smo.20230025)
Supplement: Supplementary file 1 — Supporting Information S1 [file SMO2-2-e20230025-s001.docx]

Prof. Xiaojun Peng

Editor-in-Chief,

Smart Molecules

**Re: Manuscript ID. SMO.20230025**

Dear Prof. Peng:

Thank you so much for your kind email dated on Dec. 25, 2023. I truly appreciate all you and your editorial colleagues have kindly done for our review manuscript on this exciting topic of Smart Chiral Liquid Crystal Elastomers. I also thank three respected referees for their thoughtful and encouraging comments, and appreciate the time they spent providing valuable feedback on our manuscript. We are excited to be informed that our manuscript would be recommended for publication in your esteemed journal *Smart Molecules* pending satisfactory revisions.

I have uploaded our revised manuscript.doc, revised manuscript with highlighted changes.pdf, and documents of copyright permissions. I certify that this manuscript or a related manuscript has not been published in any journals including electronic journals and computer databases of a public nature.

Our point-by-point response to the respected referees’ comments and the changes made in the revised manuscript are as follows.

**Reviewer #1:**

“*In this manuscript, the authors provide a comprehensive review of the state-of-the-art advances and emerging applications of chiral liquid crystal elastomers, focusing on different fabrication methods including surface-enforced alignment, bar coating, 3D printing, and anisotropic deswelling. They also discussed the properties and applications of CLCEs, as well as the challenges and opportunities for future development. This topic embraces optics, physics, chemistry, materials science, device engineering, and other interdisciplinary areas, therefore, this timely review would have a broad impact on a wide range of audiences. The paper is well-written, well-organized, and well-illustrated, with clear objectives, logical structure, and informative figures. The paper demonstrates a deep understanding of the underlying physics and chemistry of CLCEs, as well as the current challenges and opportunities in the field, and provides a valuable perspective for future research directions. I would strongly recommend this manuscript to be accepted for publication in* ***Smart Molecules*** *with the following satisfactory improvement.*”

Our response: We greatly appreciate the respected reviewer for her or his time and recognizing the significance of our work. This topic embraces materials science, chemistry, nanoscience, materials science, device engineering, nanotechnology, and other interdisciplinary areas. We sincerely believe that this Review would have broad impact on a wide range of audience. We also thank the respected referee for her/his precious and constructive suggestions, which greatly help us to improve our manuscript.

“*1. Authors should add a table to briefly summarize the advantages and disadvantages of each method in CLCEs at the end of the manuscript.*”

**Our response:** Per suggestion, Table 1 has been added in the revised manuscript to illustrate the mechanisms, advantages, disadvantages, application, and materials of these preparation methods suited for chiral liquid crystal elastomers, which is placed at the end of section 7. (Page 35)

“*2. The authors had better provide appropriate summary and critical discussions at the end of Section 4 and Section 5.*”

**Our response:** Thanks for your valuable suggestion. The relevant summary has been added in the revised manuscript.

“*3. Please carefully read the manuscript again, and fix the typos. For examples*

*“Since LDPE is permeable dichloromethane, diffusion and consequently continuous evaporation of the solventthrough the tube wall for two days at 45 °C allowed the CLC phase to self-assemble with a radially oriented helical pitch (Figure 10c).” solventthrough should be “solvent through”.*

**Our response:** Thanks for kind suggestion. We have thoroughly revised the manuscript and corrected typos.

**Reviewer #2**

“*In this manuscript, Prof. Feng, Prof. Wang and coworkers explore the advancements in smart chiral liquid crystal elastomers (CLCEs), focusing on their design, fabrication, and applications. CLCEs, including cholesteric and blue-phase variants, exhibit tunable reflected colors in response to external stimuli. Various fabrication methods such as surface-enforced alignment, bar coating, 3D printing, and anisotropic deswelling are discussed. The applications of smart CLCEs range from adaptive optics to soft robotics. The review anticipates that the maturation of CLCEs will lead to diverse applications, emphasizing the exploitation of properties beyond reversible color change and soft elasticity. Overall, the topic is very interesting. The discussion is sufficient. I would recommend the manuscript be accepted for publication after some minor revisions.*”

**Our response**: We greatly appreciate the respected reviewer’s time and expertise in assessing our manuscript and recognizing the significance of the work presented in the manuscript with very encouraging comments. This topic also embraces recent reaction mechanisms and strategies to synthesize and align chiral liquid crystal elastomers. Since there are no such reviews which comprehensively cover such flourishing topic in a unified approach, we sincerely feel it is the right time to assemble the scattered literature of this important research field, which is expected to provide significant impetus towards the rapid advancement of this highly important endeavor rooted from chemistry and materials science. Therefore, we sincerely believe that this Review would have broad impact on a wide range of audience. We also thank the respected referee for her/his precious and constructive suggestions, which greatly help us to improve our manuscript.

“*1. It is recommended that the advantages, the application scenarios, and the suitable chemical systems of the four methods for preparing CLCEs, including surface-enforced alignment, bar coating, 3D printing, and anisotropic deswelling, should be clearly discussed. If possible, supplementing a table would be better.*”

**Our response**: Per suggestion, Table 1 has been added in the revised manuscript to illustrate the mechanisms, advantages, disadvantages, application, and materials of these preparation methods suited for chiral liquid crystal elastomers, which is placed at the end of section 7. (Page 35)

“*2. The sizes of the chemical structures and labels in Figure 6a are too small. Also, the resolution of this figure is not high enough.*”

**Our response**: Thanks for kind suggestion. We have thoroughly revised the manuscript about figure 6a.

“*3. In each section, there could be a brief summary.*”

**Our response**: Thanks for your valuable suggestion. The relevant brief summary has been added in the revised manuscript.

“*4. All discussions are limited to CLCE-based structural colors. Actually, other CLCEs that show* *photochromic luminescence or circularly polarized luminescence have been reported. Why these materials are not included in the scope of this review should be explained in the introduction section.*”

**Our response**: Many thanks for kind suggestions. The other CLCEs that show photochromic luminescence or circularly polarized luminescence is indeed a part that cannot be ignored, which results the constructed system with diverse properties. To demonstrate the importance of this part, we conduct a detailed discussion in the conclusion section. The detailed description and relevant references have been added in the revised manuscript.

“*5. Also, why the authors choose to divide the sections based on the* *preparation method, is there any previous review that has discussed a similar topic, and what the difference and novelty of this review is, should be clearly clarified in the introduction section.*”

**Our response**: Thanks for your valuable suggestion. To date, substantial advancements have been achieved in the development of chiral liquid crystal networks, the alignment of mesogen orientations, and the construction of stimulus-responsive LCE actuators. Several excellent reviews summarizing these advancements in more detail have been reported. Recently, diverse preparation methods and applications of chiral LCEs with both structural color and polymer elasticity have developed rapidly. It is worth mentioning that since long-range ordered mesogen orientation is the basis for high-quality structural color and macroscopic deformation of chiral LCEs, different alignment methods including surface-enforced alignment, bar coating, 3D printing and anisotropic deswelling have been developed to obtain better mesogen orientation. Researchers have used different orientation strategies to achieve the preparation of chiral LCEs with stimulus-responsive structural colors, broadband spectral shifts, good mechanical properties, and large-scale processability, and identified their potential applications in biomimetic camouflage, information encryption, and programmable shape. However, there remains a notable gap in the literature, as a comprehensive review encompassing the design, properties and applications of chiral LCEs. To provide useful insights to the research community, it is necessary to summarize the recent progress related to chiral LCEs. The detailed description has been added at introduction in the revised manuscript on Page 4.

**Reviewer #3:**

**“***This is a very interesting manuscript focusing on the design, properties, and application of smart chiral liquid crystal elastomers. The authors reviewed the recent progress in the self-assembly methods of CLCEs, such as surface-enforced alignment, bar coating, 3D printing, and anisotropic deswelling, and the corresponding advantages and disadvantages are discussed. They also summarized the properties and potential applications of CLCEs, such as photonic crystals, mechanochromism, programmability, and self-healing. It demonstrates the versatility and functionality of CLCEs as soft photonic crystals with tunable structural colors, shape-morphing, and stimuli-responsive properties, which are attractive for various fields such as optics, photonics, robotics, and security. The logic of this manuscript is very clear and gives us a deeper understanding of the properties of smart CLCEs and as such satisfies the criteria of Smart Molecules. I recommend publishing this manuscript after some minor revisions.*”

**Our response:** We truly appreciate the respected referee for recognizing the novelty and significance of our work. Since there are no such reviews which comprehensively cover such flourishing topic in a unified approach, we sincerely feel it is the right time to assemble the scattered literature of this important research field, which is expected to provide significant impetus towards the rapid advancement of this highly interdisciplinary endeavor rooted from materials science and chemistry.

“*1. “The self-assembled nanostructure of Chiral liquid crystal elastomers can be dynamically controlled under external stimulation,” Chiral liquid crystal elastomers should be “chiral liquid crystal elastomers”. Please pay attention to the writing details.*”

**Our response:** Many thanks for valuable suggestions. We have thoroughly revised the manuscript and corrected typos or errors.

“*2. At Section 2 “By simply changing the external layers (PDMS or PMP)”, the abbreviation of PMP is not defined.”*

**Our response:** Thank you for kind suggestion. We have replaced "PMP" with " Methyl pentene copolymer (PMP)" in this sentence. (Page 10).

“*3. Please unify the format of the review literature according to the reference format of Smart Molecules.”*

**Our response:** Per suggestion, we have unified the format of the review literature according to the reference format of *Smart Molecules*.

“*4. It would be nice if the authors could comment on the main properties, and possible limitation in the sections of surface-enforced alignment, bar coating, 3D printing, and anisotropic deswelling.*”

**Our response:** Per suggestion, Table 1 has been added in the revised manuscript to illustrate the mechanisms, advantages, disadvantages, application, and materials of these preparation methods suited for chiral liquid crystal elastomers, which is placed at the end of section 7. (Page 35)

Furthermore, we have very carefully checked the manuscript. With the changes and point-by-point response to respected reviewers’ comments, we hope that the revised manuscript is now acceptable for the publication.

Thank you and best regards,

Ling WANG, Ph. D

Professor, School of Materials Science and Engineering,

Tianjin University, P. R. China

Homepage:[www.wanglinglab.com](http://www.wanglinglab.com)

Wei FENG, Ph. D

Professor, School of Materials Science and Engineering,

Tianjin University, P. R. China
